# *N*-Heterocyclic Carbene Coinage Metal Complexes of the Germanium-Rich Metalloid Clusters [Ge_9_R_3_]^−^ and [Ge_9_R^I^_2_]^2−^ with R = Si(*^i^*Pr)_3_ and R^I^ = Si(TMS)_3_

**DOI:** 10.3390/molecules22071204

**Published:** 2017-07-19

**Authors:** Felix S. Geitner, Michael A. Giebel, Alexander Pöthig, Thomas F. Fässler

**Affiliations:** 1 Wacker Institute for Silicon Chemistry and Department of Chemistry, Technische Universität München, Lichtenbergstraße 4, 85747 Garching, Germany; felix.geitner@mytum.de; 2 Department of Chemistry, Technische Universität München, Lichtenbergstraße 4, 85747 Garching, Germany; michael.giebel@tum.de; 3 TUM Catalysis Research Center (CRC), Ernst-Otto-Fischer Straße 1, 85747 Garching, Germany; alexander.poethig@tum.de

**Keywords:** coinage metal, NHC compounds, *Zintl* clusters, binuclear compounds

## Abstract

We report on the synthesis of novel coinage metal NHC (*N*-heterocyclic carbene) compounds of the germanium-rich metalloid clusters [Ge_9_R_3_]^−^ and [Ge_9_R^I^_2_]^2−^ with R = Si(*^i^*Pr)_3_ and R^I^ = Si(TMS)_3_. NHC^Dipp^Cu{η^3^-Ge_9_R_3_} with R = Si(*^i^*Pr)_3_ (**1**) represents a less bulky silyl group-substituted derivative of the known analogous compounds with R = Si(*^i^*Bu)_3_ or Si(TMS)_3_. The coordination of the [NHC^Dipp^Cu]^+^ moiety to the cluster unit occurs via one triangular face of the tri-capped trigonal prismatic [Ge_9_] cluster. Furthermore, a series of novel *Zintl* cluster coinage metal NHC compounds of the type (NHC*M*)_2_{η^3^-Ge_9_R^I^_2_} (R^I^ = Si(TMS)_3_ *M* = Cu, Ag and Au; NHC = NHC^Dipp^ or NHC^Mes^) is presented. These novel compounds represent a new class of neutral dinuclear *Zintl* cluster coinage metal NHC compounds, which are obtained either by the stepwise reaction of a suspension of K_12_Ge_17_ with Si(TMS)_3_Cl and the coinage metal carbene complexes NHC*M*Cl (*M* = Cu, Ag, Au), or via a homogenous reaction using the preformed bis-silylated cluster K_2_[Ge_9_(Si(TMS)_3_)_2_] and the corresponding NHC*M*Cl (*M* = Cu, Ag, Au) complex. The molecular structures of NHC^Dipp^Cu{η^3^-Ge_9_(Si(*^i^*Pr)_3_)_3_} (**1**) and (NHC^Dipp^Cu)_2_{η^3^-Ge_9_(Si(TMS)_3_)_2_} (**2**) were determined by single crystal X-ray diffraction methods. In **2**, the coordination of the [NHC^Dipp^Cu]^+^ moieties to the cluster unit takes place via both open triangular faces of the [Ge_9_] entity. Furthermore, all compounds were characterized by means of NMR spectroscopy (^1^H, ^13^C, ^29^Si) and ESI-MS.

## 1. Introduction

Polyatomic *Zintl* clusters [E_9_]^4−^ or [E_4_]^4−^ (E: tetrel element) can be extracted from the neat solids of their alkali metal salts with the compositions A_4_E_9_ and A_12_E_17_ (A = alkali metal, E = Si-Pb) and are then accessible for further reactions. The first synthesis of an organometallic *Zintl* cluster species, [Sn_9_Cr(CO)_3_]^4−^, was achieved in 1988 by treatment of the *Zintl* phase K_4_Sn_9_ with Cr(CO)_3_Mes (Mes = mesitylene) [1]. Since then, a large number of transition metal compounds containing *Zintl* cluster ligands have been reported. In various studies, precursors, especially of the late transition metals (groups 10, 11 and 12), have successfully been treated with these anionic *Zintl* clusters [2,3]. However, the addition of early transition metal fragments to *Zintl* clusters has been recently achieved [4].

Reactions of *Zintl* clusters with coinage metal precursor complexes and Zn organyls revealed that the addition of a metal atom to the bare clusters can also initiate cluster growth under formation of larger intermetalloid clusters. For example the reaction of the *Zintl* phase K_4_Ge_9_ with R_3_PCuCl (R = alkyl, aryl) at first leads to [(R_3_P)Cu(η^4^-Ge_9_)]^3−^, but the subsequent substitution of the PR_3_ group by a second [Ge_9_] cluster unit results in the coinage metal-bridged dimeric species [(η^4^-Ge_9_)Cu(η^1^-Ge_9_)]^7−^ [5]. Furthermore, reactions of tetrel clusters with Ph_2_Zn yield compounds of the shape [E_9_ZnPh]^3−^ (E: Si-Pb) [6], and similar reactions with Cp_2_Zn afford heteroatomic *closo*-clusters [Ge_9_Zn]^2−^, which, due to their electron donor and acceptor capabilities, allow for inter-cluster growth to form ${}_{\infty }^{1}$[{Zn[(η^4^:η^1^-Ge_9_)]}^2−^] polymers [7]. In the case of the clusters of the heavier homologues Sn and Pb, the migration of Cu^+^ into the polyhedral clusters under the loss of the original ligand sphere and formation of the endohedrally filled [Cu@E_9_]^3−^ (E = Sn, Pb) clusters was observed for CuMes [8]. By contrast, the reaction of CuMes with the smaller tetrahedral *Zintl* clusters [E_4_]^4−^ leads to the mesitylcopper complexes [(MesCu)_2_(η^3^-E_4_)]^4−^ (E = Si, Ge) [9,10]. Additionally, larger intermetalloids [Au_3_Ge_18_]^5−^ and [Au_3_Ge_45_]^4−^ were obtained in the reaction of bare [Ge_9_]^4−^ clusters with (PPh_3_)AuCl [11,12]. In recent studies, we introduced *N*-heterocyclic carbene ligands (NHCs) into the *Zintl* cluster chemistry by treating coinage metal carbene compounds NHC^Dipp^*M*Cl (*M* = Cu, Ag, Au) with bare or silylated *Zintl* clusters. The reaction of NHC^Dipp^*M*Cl (*M* = Cu, Ag, Au) with [Sn_9_]^4−^ in NH_3_ (l) yielded a series of anionic *Zintl* cluster coinage metal NHC compounds [NHC^Dipp^*M*(η^4^-Sn_9_)]^3−^ (*M* = Cu, Ag, Au). For [NHC^Dipp^Ag(η^4^-Sn_9_)]^3^**^−^** a subsequent reaction takes already place at −70 °C, giving [(η^4^-Sn_9_)Ag(η^1^-Sn_9_)]^7−^ via a nucleophilic attack of a second [Sn_9_]^4−^ cluster unit at the Ag^+^ center, under cleavage of the Ag-NHC bond [13]. Thus, the obtained species can be regarded as low-temperature intermediates on the way to larger intermetalloid compounds. These products show that bare tetrel *Zintl* clusters can act as ligands in organometallic complexes, and that subsequent reactions of such species can lead to larger intermetalloids. However, in some cases, unforeseen oxidation reactions of the bare *Zintl* clusters can also occur, and it should be mentioned that, due to their highly negative charges, homogenous reactions of bare *Zintl* clusters usually require highly polar solvents (ethylendiamine, dmf, NH_3_(l)).

Much less reactive germanide *Zintl* clusters can be obtained by silyl substitution of the [Ge_9_]^4−^ clusters. The resulting bis- and tris-silylated cluster species [Ge_9_R_2_]^2−^ (R = Si(TMS)_3_) [14] and [Ge_9_R_3_]**^−^** (R: various silyl groups) [15,16,17,18,19,20] are, due to their lower charge, soluble in common solvents (acetonitrile, thf, toluene). Subsequent reactions of the tris-silylated cluster [Ge_9_R_3_]**^−^** (R: Si(TMS)_3_) with coinage metal complexes, in most cases coinage metal phosphine complexes, commonly yield coinage metal-bridged cluster dimers, under the loss of the original ligand sphere of the transition metal [19,21,22]. However, there are also a few reports in which the Cu-phosphine bond is retained [18,23]. The reaction of coinage metal NHC complexes such as NHC^Dipp^*M*Cl (*M* = Cu, Ag, Au) with tris-silylated clusters [Ge_9_R_3_]**^−^** (R = Si(TMS)_3_, Si(*^i^*Bu)_3_) leads to NHC^Dipp^*M*{η^3^-Ge_9_(Si(TMS)_3_)_3_} (*M* = Cu, Ag, Au) [24] or NHC^Dipp^Cu{η^3^-Ge_9_(Si(*^i^*Bu)_3_)_3_} [19]. These uncharged complexes readily dissolve in rather non-polar solvents such as thf or toluene. Furthermore, these species are stable at room temperature, except for NHC^Dipp^Ag{η^3^-Ge_9_(Si(TMS)_3_)_3_}, for which a transformation reaction in thf solution under formation of [Ag{η^3^-Ge_9_(Si(TMS)_3_)_3_}_2_][Ag(NHC^Dipp^)_2_] is observed within seven days [24].

In continuation of our work, we examined the reactivity of coinage metal carbene compounds towards silylated *Zintl* clusters and studied the impact of a tris-silylated cluster ligand, bearing the sterically less bulky silyl group [Si(*^i^*Pr)_3_]^+^ on the structure of the resulting Cu-NHC complex. Furthermore, we investigated the reactivity of coinage metal carbene compounds towards solutions obtained from the silylation of the *Zintl* phase K_12_Ge_17_ instead of the previously used phase K_4_Ge_9_. Finally, we studied the reactivity of NHC*M*Cl (*M* = Cu, Ag, Au) complexes towards the bis-silylated cluster compound [Ge_9_{Si(TMS)_3_}_2_]^2−^.

## 2. Results and Discussion

**Scheme 1:**
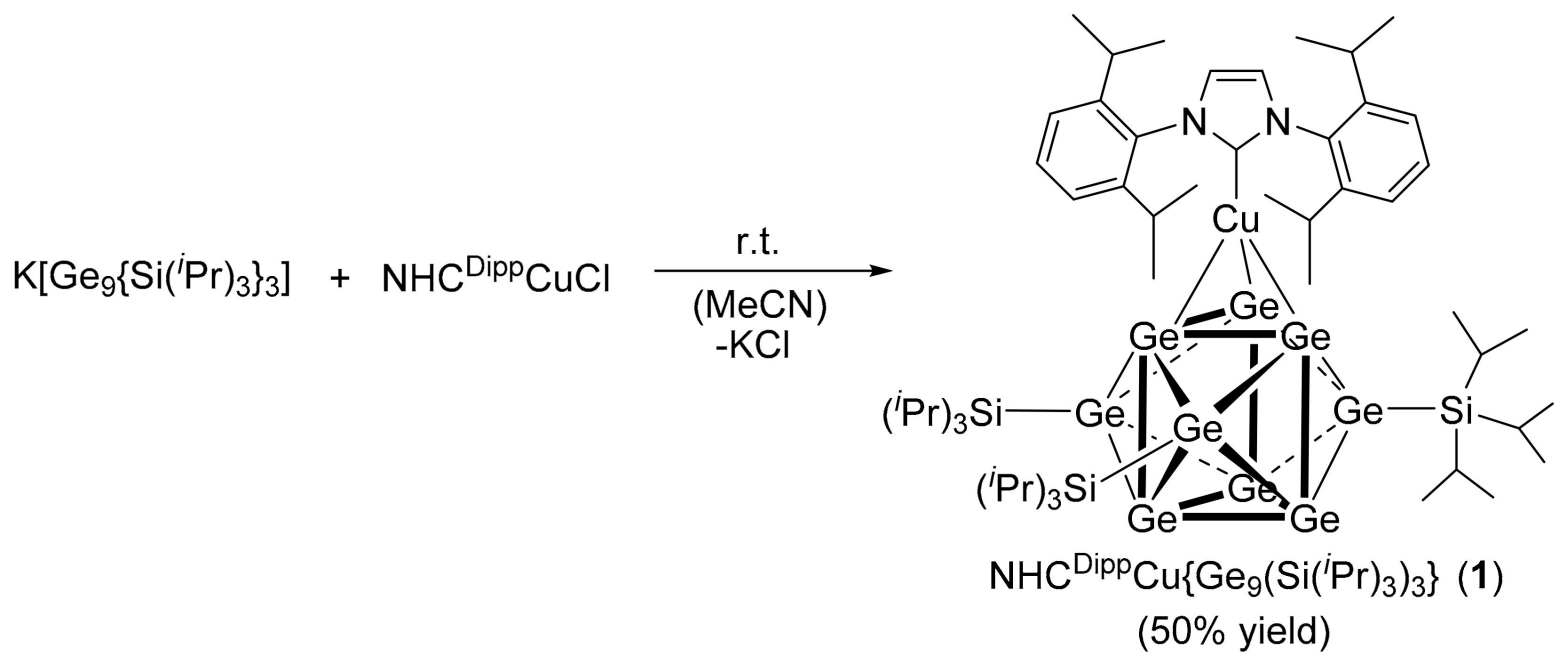
Synthesis of compound **1** from K[Ge_9_{Si(*^i^*Pr)_3_}_3_] and NHC^Dipp^CuCl.

In analogy to the synthesis of NHC^Dipp^Cu{η^3^-Ge_9_R_3_}, comprising the bulky silyl groups [Si(*^i^*Bu)_3_]^+^ or [Si(TMS)_3_]^+^, we prepared the novel *Zintl* cluster coinage metal NHC complex with the less bulky silyl group [Si(*^i^*Pr)_3_]^+^ (**1**) by reacting the respective silylated cluster species [Ge_9_{Si(*^i^*Pr)_3_}_3_]^−^ with NHC^Dipp^CuCl in acetonitrile (Scheme 1). ^1^H-NMR studies reveal a signal ratio of 3:1 for the signals assigned to the *^i^*Pr substituents (silyl groups) and the NHC^Dipp^ ligand. Remarkably, the proton signals of the isopropyl groups (CH_3_ and CH) have identical chemical shift values (*pseudo*-singlet), independent of the applied solvent (C_6_D_6_, thf-*d*_8_), as also confirmed by 2D-NMR investigations (HSQC), in which the protons can be assigned to the carbon atom they are bound to (in the ^13^C-NMR spectrum the signals of the CH_3_ and the CH groups of the isopropyl units are split; Supporting Information; Figure SI 2 and Figure SI 3).

**Figure 1:**
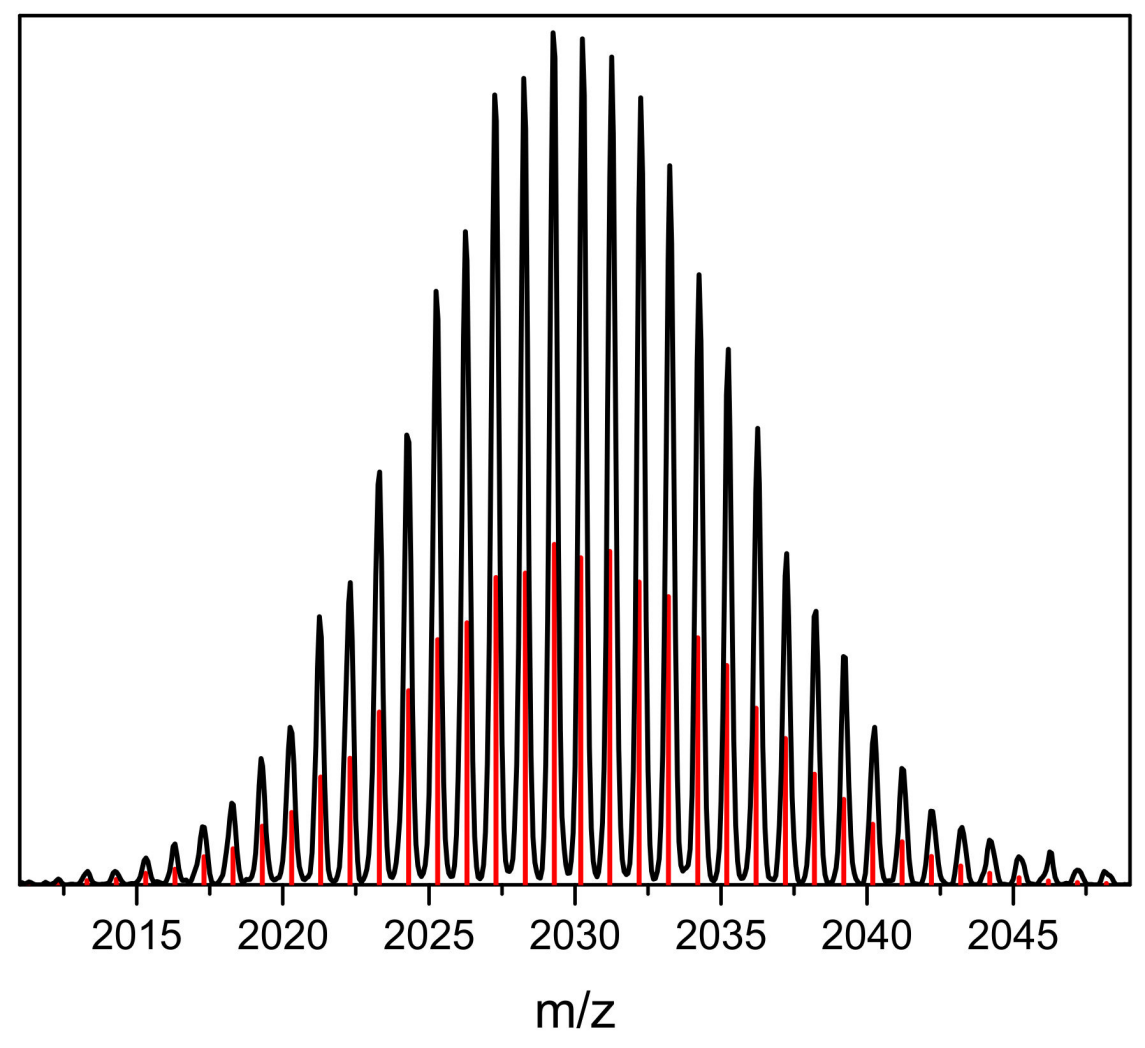
Selected area of the ESI-MS spectrum obtained by examination of a thf solution of compound **1**. The mass spectrum was acquired in positive ion mode (4500 V, 300 °C), monitoring **1** with an additionally attached [Cu-NHC]^+^ moiety at *m*/*z* 2029.3 [(NHC^Dipp^Cu)_2_{η^3^-Ge_9_(Si(*^i^*Pr)_3_)_3_}]^+^. The calculated pattern with the characteristic isotope distribution is shown below as red bars.

In the ESI-MS experiment carried out with athf solution of **1**, a signal is detected at *m*/*z* 2029.3 [(NHC^Dipp^Cu)_2_{η^3^-Ge_9_(Si(*^i^*Pr)_3_)_3_}]^+^ in the positive ion mode, matching the mass obtained by the addition of a second [Cu-NHC^Dipp^]^+^ fragment to **1** (Figure 1).

**Figure 2:**
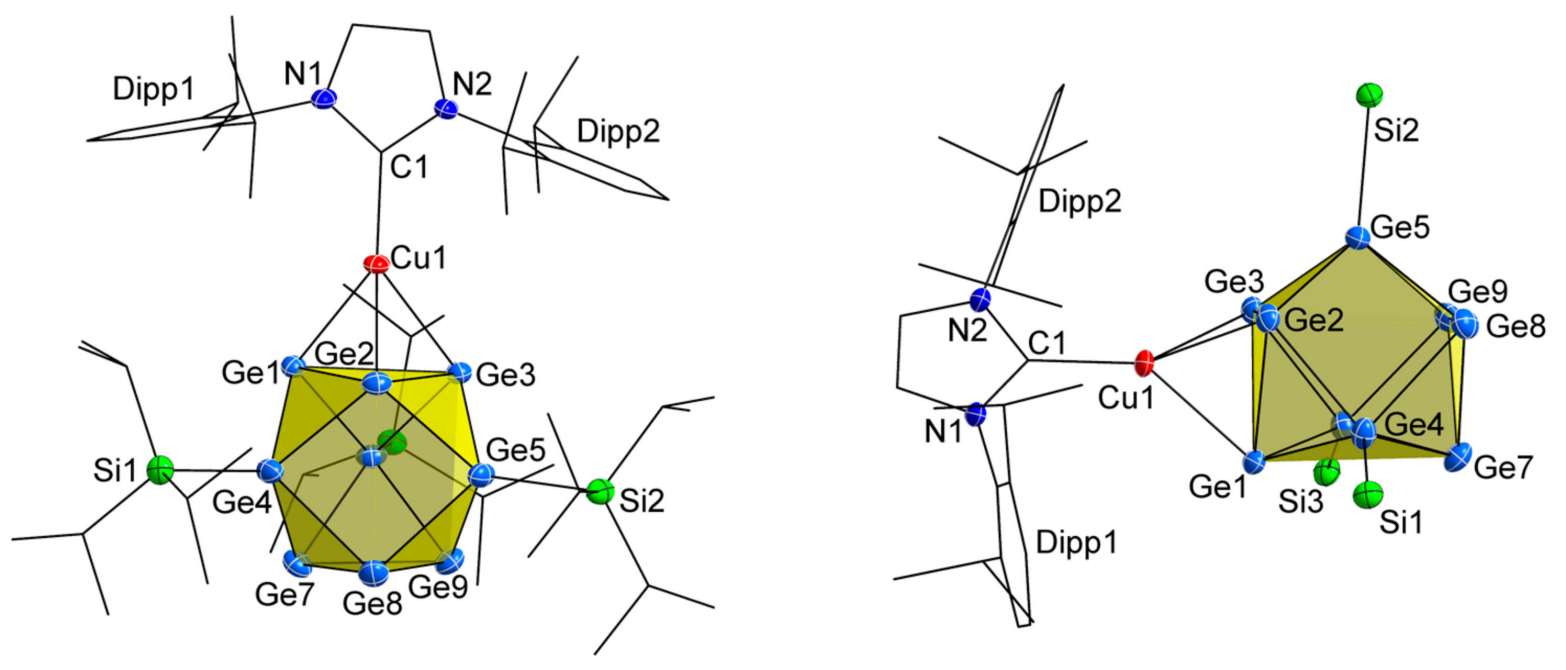
Two perspective views of the molecular structure of compound **1**. Displacement ellipsoids are shown at a 50% probability level. For clarity, hydrogen atoms and co-crystallized toluene molecules are omitted. To the right: Side view of **1**, revealing a nearly linear coordination of Cu^+^ by NHC^Dipp^ and the center of one triangular plane of [Ge_9_{Si(*^i^*Pr)_3_}_3_]^−^. Isopropyl groups of the silyl substituents of the cluster are omitted. Selected bond lengths and angles are summarized in Table 1 and the Supporting Information (Table SI 1).

Recrystallization of **1** from toluene at −40 °C resulted in red crystals, suitable for single crystal X-ray diffraction. The purity of the sample was confirmed by elemental analysis of the red solid obtained by grinding the crystals. In **1**, the [NHC^Dipp^Cu]^+^ moiety is coordinated to the tri-capped trigonal prismatic [Ge_9_{Si(*^i^*Pr)_3_}_3_]^−^ cluster via one of the triangular faces in a η^3^-fashion with a mean Cu-Ge distance of 2.5328(9) Å (Figure 2; bond lengths are summarized in Table 1).

**Figure 3:**
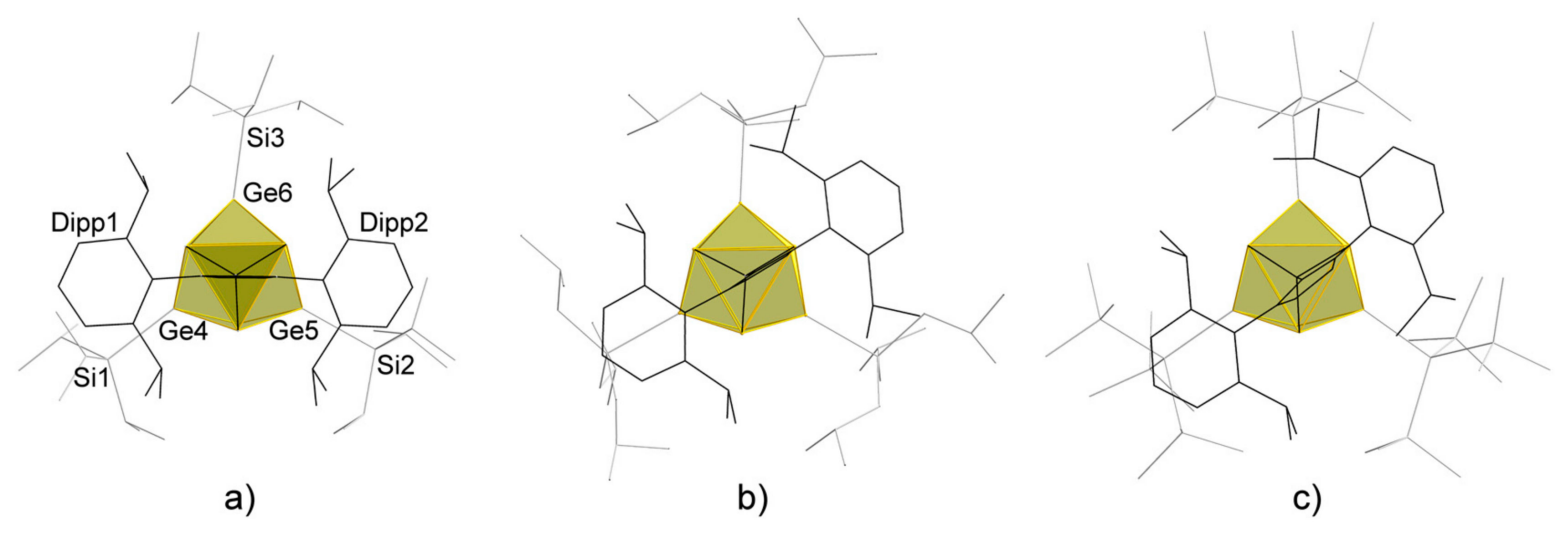
Top views of NHC^Dipp^Cu{η^3^-Ge_9_R_3_}. The silyl groups connected to the cluster are shown as grey wire frames. (**a**) R = Si(*^i^*Pr)_3_, **1**; (**b**) R = Si(*^i^*Bu)_3_ [19] and (**c**) R = Si(TMS)_3_ [24].

The Cu^+^ center is linearly coordinated by the NHC and *Zintl* cluster ligands (177.7°; Table 1), and the Cu-C1 (C1: carbene carbon) bond length of 1.951(3) Å is in the range of previously reported data [24]. However, the orientation of the NHC^Dipp^ ligand at Cu^+^ with respect to the silyl substituents of the *Zintl* cluster in **1** differs from that in NHC^Dipp^Cu{η^3^-Ge_9_R_3_} with the bulkier ligands [Si(*^i^*Bu)_3_]^+^ or [Si(TMS)_3_]^+^ (Figure 3b,c). In **1**, the NHC^Dipp^ ligand shows a more ecliptic arrangement towards two of the [Si(*^i^*Pr)_3_]^+^ groups attached to the [Ge_9_] unit (Figure 3a), whereas in the analogous compounds with the larger silyl groups one di-isopropyl (Dipp) wingtip of the NHC ligand shows a staggered arrangement towards two of silyl groups, and consequently the other wingtip is situated directly above the third silyl substituent (Figure 3b,c). As a result, the coordination of Cu^+^ by NHC^Dipp^ ligand and silylated cluster deviates less from linearity in **1,** than in NHC^Dipp^Cu{η^3^-Ge_9_R_3_} with R = Si(*^i^*Bu)_3_ or Si(TMS)_3_.

In order to explore other sources for germanium clusters, we investigated the synthesis of silylated clusters by using the *Zintl* phase K_12_Ge_17_ as cluster source. This phase contains [Ge_9_]^4−^ clusters as well as tetrahedral [Ge_4_]^4−^ units. Therefore, analogous reactions might lead to the extraction of other silylated cluster species. In a previous study, we were able to isolate [Si_4_]^4−^ clusters as MesCu complexes from a starting material of the composition K_12_Si_17_, carrying out reactions in NH_3_ (l) [9]. In the current study, we found that the heterogenous reaction of K_12_Ge_17_ with 6 eq. of Si(TMS)_3_Cl in acetonitrile also leads to a deep red suspension upon stirring over night at room temperature, in analogy to the silylation reaction of K_4_Ge_9_ [16].

After filtration, the addition of a solution of NHC^Dipp^CuCl in acetonitrile to this deep red filtrate immediately led to the formation of a brownish precipitate, which was isolated by filtering off the supernatant solution. The solid was then dissolved in toluene, and the mixture was filtered to remove KCl formed during the reaction. Subsequently, the solvent was removed in vacuo yielding the crude product as a brown solid. ^1^H-NMR measurements indicated the attachment of [NHC^Dipp^Cu]^+^ to [Ge_9_] by a significant divergence of the doublets assigned to the methyl groups of the diisopropylphenyl wingtips of the NHC ligand, which had previously been observed upon similar reactions [19,24]. Furthermore, the 1:1 signal ratio of the signals assigned to the protons of the hypersilyl groups and those of the NHC^Dipp^ ligand, as well as the poor solubility of the product in acetonitrile and its good solubility in non-polar solvents such as thf and toluene suggested the presence of an uncharged species according to a composition of (NHC^Dipp^Cu)_2_{η^3^-Ge_9_(Si(TMS)_3_)_2_} (**2**).

**Figure 4:**
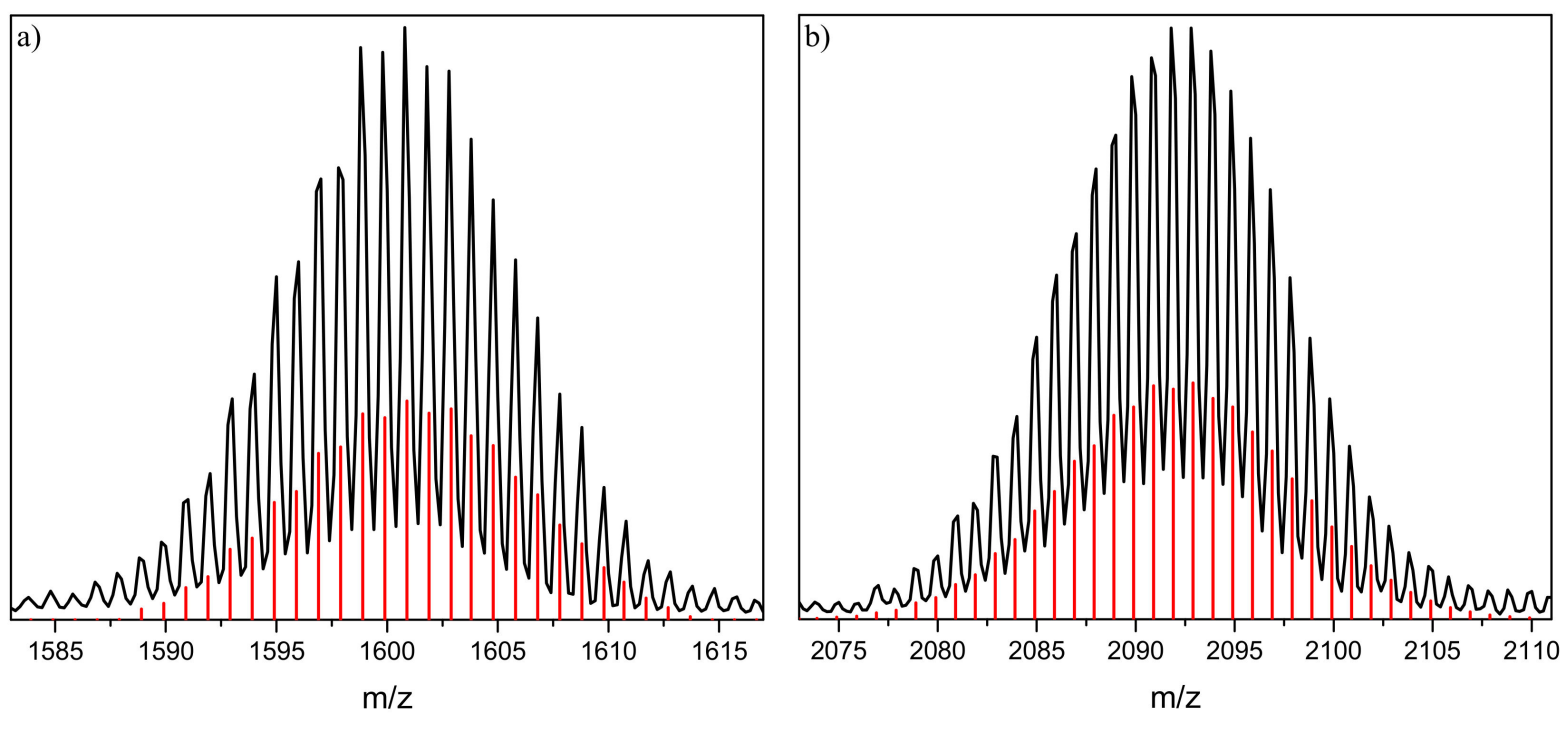
Selected areas of the ESI-MS spectrum obtained by examination of an acetonitrile solution of compound **2**. (**a**) Negative ion mode (4500 V, 300 °C), monitoring **2** with a cleaved [Cu-NHC]^+^ moiety at *m*/*z* 1600.8 [NHC^Dipp^Cu{Ge_9_(Si(TMS)_3_)_2_}]^−^ and (**b**) positive ion mode (6000 V, 300 °C), monitoring **2** with an attached potassium cation at *m*/*z* 2092.8 [(NHC^Dipp^Cu)_2_{Ge_9_(Si(TMS)_3_)_2_}K]^+^. The simulated mass spectra with the corresponding isotope distribution are shown below as red bars.

In ESI-MS examination of an acetonitrile solution of the product, signals were detected at *m*/*z* 1600.8 in the negative ion mode and at *m*/*z* 2092.8 in the positive ion mode, corresponding to [NHC^Dipp^Cu{Ge_9_(Si(TMS)_3_)_2_}]^−^ or [(NHC^Dipp^Cu)_2_{Ge_9_(Si(TMS)_3_)_2_}K]^+^, respectively (Figure 4). In combination with the characteristic isotope distribution, these observations were a strong indication for the existence of **2**.

Single crystals of **2** suitable for an X-ray diffraction structure determination were obtained by recrystallization from toluene at −40 °C. The analysis of the obtained diffraction data confirmed the assumptions derived from the NMR and ESI-MS experiments, and revealed the first dinuclear *Zintl* cluster coinage metal NHC complex (NHC^Dipp^Cu)_2_{η^3^-Ge_9_R^I^_2_} (R^I^ = Si(TMS)_3_) (**2**). The central [Ge_9_] unit adopts the shape of a distorted, *C*_2*v*_-symmetric, monocapped square antiprism, in which two opposite Ge atoms (Ge6 and Ge8) of the open square (Ge6-Ge7-Ge8-Ge9) carry the silyl groups. The deviation from *C*_4*v*_-symmetry is expressed by the ratio of the diagonal lengths of the open square *d*(Ge7-Ge9)/*d*(Ge6-Ge8) = 1.11 (with *d*2/*d*1 = 1 for a perfect *C*_4*v*_-symmetry), and the deviation from *D*_3*h*_-symmetry of a tri-capped trigonal prism is revealed by the significantly different three prism heights *h*(Ge2-Ge3) = 2.9003(3) Å, *h*(Ge4-Ge5) = 2.9054(3) Å and *h*(Ge7-Ge9) = 3.6969(4) Å, which are supposed to be of equal length for perfect *D*_3*h*_-symmetry.

**Figure 5:**
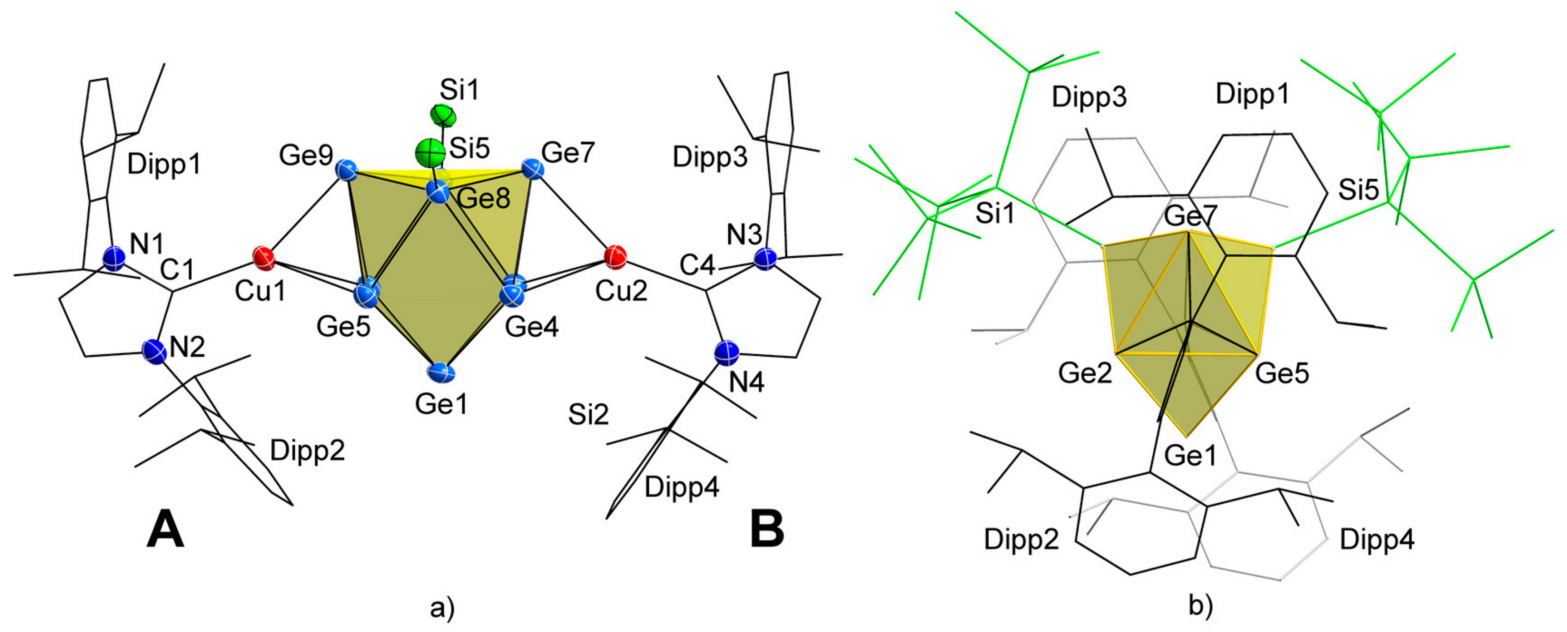
Two perspectives of the molecular structure of compound **2**. (**a**) Front view of **2** revealing the *C*_2*v*_-symmetry of the cluster. Displacement ellipsoids are shown at a 50 % probability level. For clarity, hydrogen atoms, co-crystallized toluene molecules, and the TMS groups are omitted. The two [NHC^Dipp^Cu]^+^ moieties are labelled as **A** and **B**; (**b**) View along the Cu1–Cu2 vector showing the orientation of the two NHC^Dipp^ moieties and the silyl substituents of the [Ge_9_] cluster. All ligands are pictured as wire frames. For clarity, the NHC^Dipp^ ligand in the background is pictured in grey and the silyl groups are shown in green. Selected bond lengths and angles are summarized in Table 2 and in the Supporting Information (Table SI 2).

The two [NHC^Dipp^Cu]^+^ moieties coordinate in a η^3^-fashion to the two opposed trigonal faces of the [Ge_9_] unit adjacent to the uncapped rectangle (Ge6-Ge7-Ge8-Ge9) and include the germanium atoms (Ge7 or Ge9), which do not bind to a silyl group (Figure 5). Hence, the interaction between the bis-silylated [Ge_9_] cluster and the two [NHC^Dipp^Cu]^+^ moieties (**A** and **B**; Figure 5a) in **2** mirrors the coordination mode, which has previously been observed in *Zintl* cluster coinage metal NHC compounds of tris-silylated [Ge_9_] cluster species. The mean Cu-Ge distances *d*_mean_(Cu-Ge) in **2** (2.513(1) Å for **A** and 2.509(1) Å for **B**; Figure 5a), as well as the Cu-C_Carbene_ distances (1.941(5) Å for **A** and 1.913(5) Å for **B**), are in good agreement with those observed in **1** and in [NHC^Dipp^Cu{η^3^-Ge_9_R_3_}] (R = Si(*^i^*Bu)_3_, Si(TMS)_3_) [19,24]. However, the coordination of Cu1 and Cu2 by NHC and *Zintl* cluster deviates more from linearity (162.88(2)° for **A** and 163.92(2)° for **B**; Table 2) than that in **1** (Table 1). Furthermore, the two NHC^Dipp^ moieties show a staggered orientation towards themselves and the silyl groups (Figure 5b). Both of these observations can be explained by interactions between the TMS groups of the hypersilyl ligands with the Dipp wingtips of the NHC ligands.

An investigation of the deep red solution, obtained by silylation of K_12_Ge_17_ (K_12_[Ge_9_][Ge_4_]_2_) with 6 eq. Si(TMS)_3_Cl prior to the addition of NHC^Dipp^CuCl by means of ^1^H and ^29^Si NMR spectroscopy revealed, that the bis-silylated cluster [Ge_9_{Si(TMS)_3_}_2_]^2−^ is formed as the main product, despite the excess of Si(TMS)_3_Cl used (assuming that only nine-atomic germanide clusters would react with Si(TMS)_3_Cl). For K_4_Ge_9_, this reaction would exclusively yield the tris-silylated cluster [Ge_9_{Si(TMS)_3_}_3_]^−^. Thus, we assume that the [Ge_4_]^4−^ clusters also partially react with Si(TMS)_3_Cl, which leads to side products, as is obvious from ^1^H and ^29^Si-INEPT NMR experiments (Supporting Information; Figure SI 19 and Figure SI 20).

**Scheme 2:**
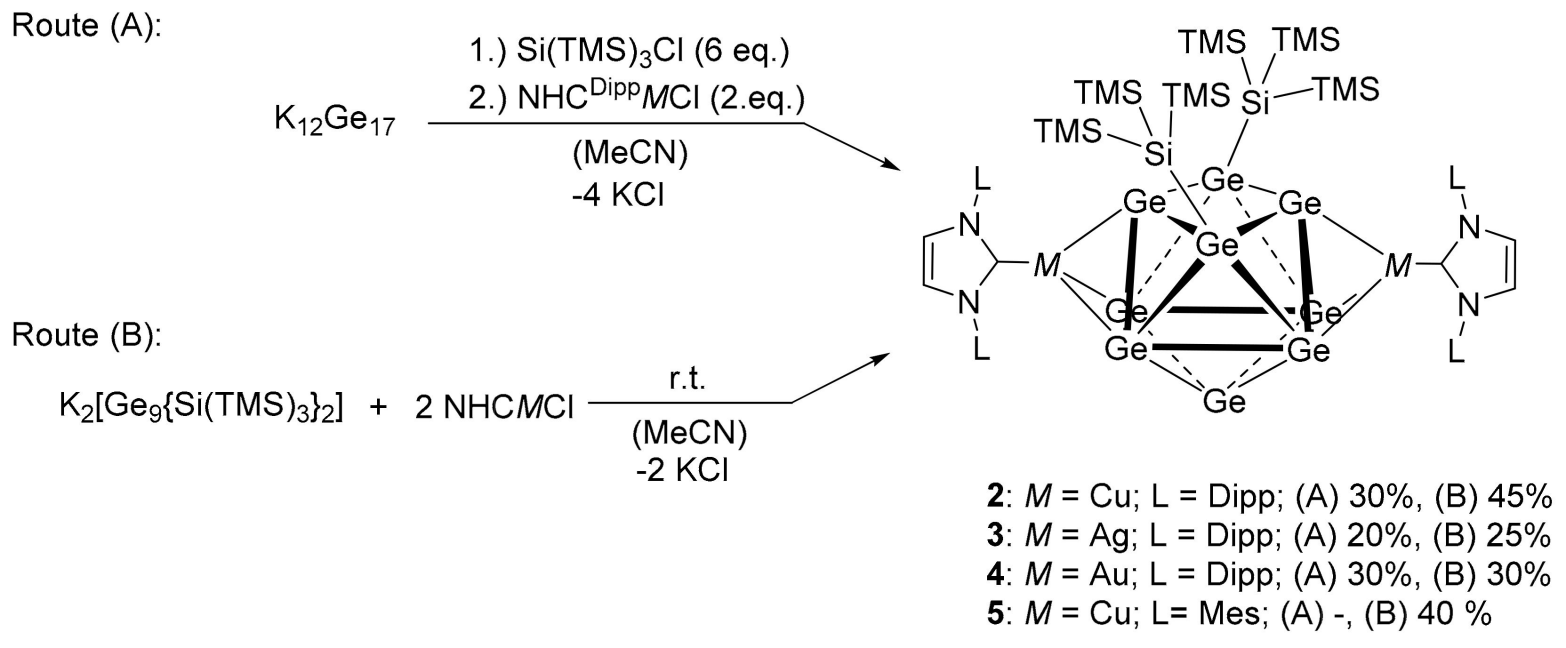
Syntheses of binuclear *Zintl* cluster coinage metal NHC compounds **2**–**5** via different routes (A or B). Wingtip substituents (L) of the NHC ligands are abbreviated as Dipp = diisopropylphenyl or Mes = mesityl. The product yields obtained by the different synthesis methods are given as (A) (product yield of route A) or (B) (product yield of route B).

**Figure 6:**
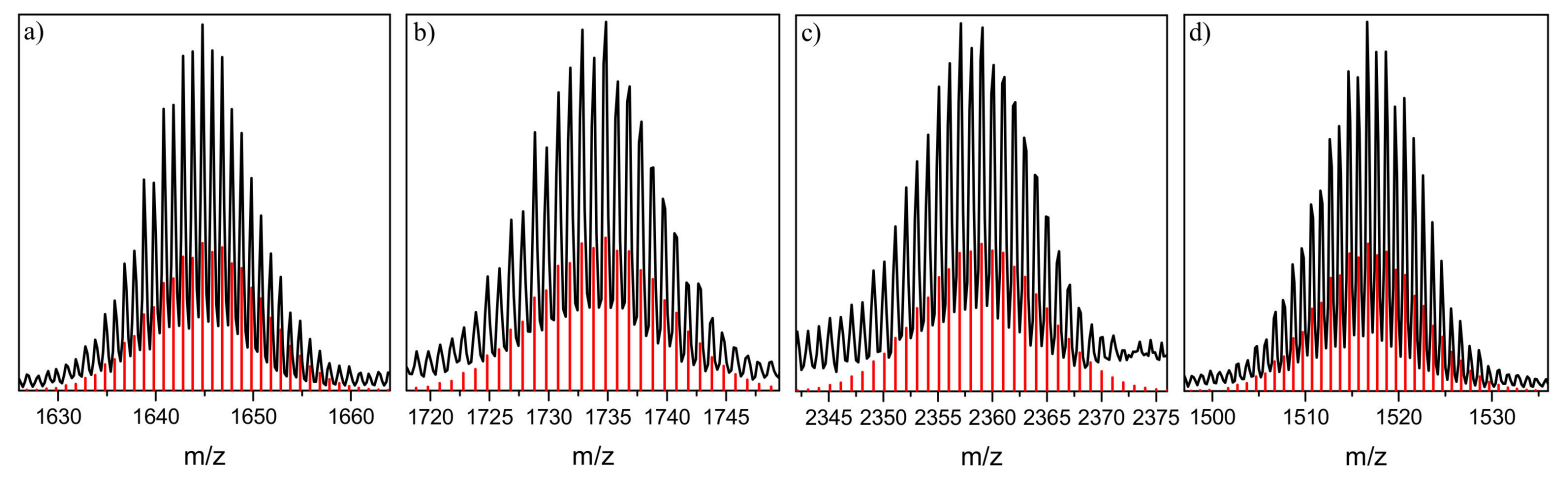
Selected areas of the ESI-MS spectra obtained by examination of a solution of (**a**) compound **3** in acetonitrile, (**b**,**c**) compound **4** in thf and (**d**) compound **5** in acetonitrile. (**a**) Negative ion mode (4500 V, 300 °C) monitoring **3** with a cleaved [Ag-NHC]^+^ moiety at *m*/*z* 1644.8 [NHC^Dipp^Ag{Ge_9_(Si(TMS)_3_)_2_}]^−^. (**b**) Negative ion mode (4500 V, 300 °C), monitoring **4** with a cleaved [Au-NHC]^+^ moiety at *m*/*z* 1734.8 [NHC^Dipp^Au{Ge_9_(Si(TMS)_3_)_2_}]^−^ and (**c**) positive ion mode (6000 V, 300 °C), monitoring **4** with an attached potassium cation at *m/z* 2359.0 [(NHC^Dipp^Au)_2_{Ge_9_(Si(TMS)_3_)_2_}K]^+^. (**d**) Negative ion mode (4500 V, 300 °C) monitoring **5** with a cleaved [Cu-NHC]^+^ moiety at *m/z* 1516.6 [NHC^Mes^Cu{Ge_9_(Si(TMS)_3_)_2_}]^−^. The simulated mass spectra with the corresponding isotope distribution are shown below as red bars.

Compound **2** can also be prepared, using the bis-silylated cluster [Ge_9_{Si(TMS)_3_}_2_]^2−^ [14] as a starting material. The addition of an acetonitrile solution of NHC^Dipp^CuCl (2 eq.) to a solution of [Ge_9_{Si(TMS)_3_}_2_]^2−^ in acetonitrile gives analytically pure **2** in good yield (45%, Scheme 2). Further experiments with NHC^Dipp^*M*Cl (*M* = Ag, Au) yielded similar products (NHC^Dipp^*M*)_2_{η^3^-Ge_9_R_2_} (*M* = Ag (**3**), Au (**4**)) according to NMR data. Furthermore, adducts of compounds **3** and **4** were detected in ESI-MS experiments (Figure 6).

The possibility to also introduce other NHC ligands by this method is shown by the reaction of the bis-silylated cluster [Ge_9_{Si(TMS)_3_}_2_]^2−^ with NHC^Mes^CuCl, bearing slightly smaller mesityl wingtip substituents. The obtained product (NHC^Mes^Cu)_2_{η^3^-Ge_9_(Si(TMS)_3_)_2_} (**5**) was characterized by NMR spectroscopy and ESI-MS (adduct with a cleaved [Cu-NHC]^+^ moiety was detected; Figure 6). Interestingly, the reaction of equimolar amounts of [Ge_9_{Si(TMS)_3_}]^2−^ and NHC^Dipp^CuCl always yielded mixtures of **2** and unreacted [Ge_9_{Si(TMS)_3_}]^2−^. Thus, the introduction of only one [NHC^Dipp^Cu]^+^ moiety remains an open challenge.

## 3. Materials and Methods

### 3.1. General

All manipulations were performed under oxygen-free, dry conditions in an argon atmosphere using standard Schlenk or glove box techniques. Glassware was dried prior to use by heating it in vacuo. The solvents used were obtained from an MBraun Grubbs apparatus. All other commercially available chemicals were used without further purification. K_4_Ge_9_ was prepared by fusion of stoichiometric amounts of the elements in stainless-steel tubes at 650 °C, and K_12_Ge_17_ was synthesized by fusion of stoichiometric amounts of the elements in tantalum containers at 800 °C. 1,3-Bis(2,6-diisopropylphenyl)imidazolium chloride, 1,3-dimesitylimidazolium chloride and the corresponding coinage metal halide complexes as well as K_2_[Ge_9_R^I^_2_] (R^I^ = Si(TMS)_3_) and K[Ge_9_R_3_] (R = Si(*^i^*Pr)_3_) were synthesized according to modified literature procedures [14,16,25,26,27,28]. All filtrations performed within this work were carried out using Whatman filter papers.

### 3.2. Single Crystal Structure Determination

The air- and moisture-sensitive crystals of **1** and **2** were transferred from the mother liquor into cooled perfluoroalkylether oil under a cold stream of N_2_ gas. For diffraction data collection, the single crystals were fixed on a glass capillary and positioned in a 150 K (**1**) or 100 K (**2**) cold N_2_ gas stream using the crystal cap system. Data collection was performed with a Bruker AXS D8 diffractometer (Mo-*Kα* radiation) (**2**) or a STOE StadiVari (Mo-*Kα* radiation) diffractometer equipped with a DECTRIS PILATUS 300K detector (**1**). Structures were solved by Direct Methods (SHELXS-2014) and refined by full-matrix least-squares calculations against *F*^2^ (SHELXL-2014) [29]. The positions of the hydrogen atoms were calculated and refined using a riding model. Unless stated otherwise, all non-hydrogen atoms were treated with anisotropic displacement parameters. The supplementary crystallographic data for this paper have been deposited with the Cambridge Structural database and are available free of charge via www.ccdc.cam.ac.uk/data_request/cif. The crystallographic data for compounds **1** and **2** are summarized in Table 3. In compound **1**, the electron density of a disordered toluene molecule was taken care of by the PLATON squeeze function [30]. Furthermore, some reflections were affected by the beamstop, and therefore they were excluded for refinement. In compound **2** one of the hypersilyl substituents is disordered and was refined on split positions. Furthermore, the electron density of a disordered toluene molecule was taken care of by the PLATON squeeze function [30].

### 3.3. NMR Spectroscopy

Sample preparation was performed in a glove box. NMR spectra were measured on a Bruker Avance Ultrashield 400 MHz spectrometer. The ^1^H- and ^13^C-NMR spectra were calibrated using the residual proton signal of the used deuterated solvents [31]. Chemical shifts are reported in parts per million (ppm) relative to TMS, with the residual solvent peak serving as internal reference. Abbreviations for signal multiplicities are: singlet (s), doublet (d), triplet (t), heptet (hept), multiplet (m). The evaluation of the spectra was carried out using the MestReNova program.

### 3.4. Electron Spray Ionization Mass Spectrometry (ESI-MS)

The sample preparation for the ESI-MS experiments was done in a glove box. ESI MS analyses were performed on a Bruker Daltronic HCT mass spectrometer (dry gas temperature: 300 °C; injection speed 240 µL/s), and the data evaluation was carried out using the Bruker Compass Data Analysis 4.0 SP 5 program (Bruker, Bremen, Germany). Spectra were plotted using OriginPro2016G (Origin Lab) and Excel 2016 (Microsoft).

### 3.5. Elemental Analyses (EA)

Elemental analyses were carried out in the micro-analytical laboratory of the Chemistry Department of Technische Universität München. Analyses of C, H, N were performed in a combustion analyzer (elementar vario EL, Bruker).

### 3.6. Syntheses

#### NHC^Dipp^Cu{η^3^-Ge_9_(Si(*^i^*Pr)_3_)_3_} (**1**)

K_4_Ge_9_ (121 mg, 0.150 mmol) was treated with an acetonitrile solution (3 mL) of Si(*^i^*Pr)_3_Cl (87 mg, 0.450 mmol). A deep red reaction mixture was obtained after stirring at r.t. over night. The suspension was filtered to remove remaining solids, and a solution of NHC^Dipp^CuCl (73 mg, 0.150 mmol, 1 eq.) in acetonitrile (1.5 mL) was added, which led to the immediate formation of an orange precipitate. The supernatant solution (slightly orange) was filtered off, and the residue was washed with acetonitrile (2 mL). After removal of the solvent in vacuo, the solids were dissolved in toluene (1 mL) and filtered to remove KCl formed upon the reactions. The sample was stored in a freezer at −40 °C for crystallization yielding the pure product as red block-shaped crystals (116 mg, 50%), suitable for single crystal X-ray diffraction.

**^1^H-NMR (400 MHz, 298 K, C_6_D_6_):** δ[ppm] = 7.33–7.27 (m, 2H, CH_Ph(p)_), 7.18 (m, 4H, CH_Ph(m)_)*, 6.26 (s, 2H, CH_Im_), 2.76 (hept, *^3^J*_HH_ = 6.4 Hz, 4H, CH*_i_*_Pr_), 1.58 (d, *^3^J*_HH_ = 6.9 Hz, 12H, Me*_i_*_Pr_), 1.27 (*pseudo*-s, 63H, Me*_i_*_Pr(silyl)_ + CH*_i_*_Pr(silyl)_), 1.07 (d, *^3^J*_HH_ = 6.9 Hz, 12H, Me*_i_*_Pr_).

**^13^C-NMR (101 MHz, 298 K, C_6_D_6_):** δ[ppm] = 145.67 (s, C_Ph(*i*Pr)_), 135.27 (s, C_Ph_N), 130.61 (s, CH_Ph(p)_), 124.36 (s, CH_Ph(m)_), 122.01 (s, CH_Im_), 29.08 (s, CH*_i_*_Pr_), 25.15 (s, Me*_i_*_Pr_), 24.82 (s, Me*_i_*_Pr_), 20.97 (s, Me*_i_*_Pr(silyl)_), 15.76 (s, CH*_i_*_Pr(silyl)_).

**^29^Si-INEPT NMR: (79 MHz, 298 K, C_6_D_6_):** δ[ppm] = 38.62 (s, Si_Ge9_).

**ESI-MS (positive mode, 4500 V, 300 °C):** 2029.3 [(NHC^Dipp^Cu)_2_{η^3^-Ge_9_(Si(*^i^*Pr)_3_)_3_}]^+^.

**Elemental analysis:** Anal. calcd. for C_54_H_99_Ge_9_Cu_1_N_2_Si_3_: C, 41.1; H, 6.3; N, 1.8; found: C, 40.7; H, 6.2; N, 1.8.

[*] Signal overlaps with the residual solvent signal of C_6_D_6_. Therefore, no multiplicity of the signal or a certain area for a multiplet could be determined. The integral of the signal is assumed to be 4, according to the 4 protons in *meta*-position at the phenyl ring causing this signal.

#### (NHC^Dipp^Cu)_2_{η^3^-Ge_9_(Si(TMS)_3_)_2_} (**2**)

Route A:

K_12_Ge_17_ (204.5 mg, 0.120 mmol) was treated with Si(TMS)_3_Cl (204.0 mg, 0.720 mmol) in a heterogeneous reaction in acetonitrile (2 mL) to form a deep red solution upon stirring at r.t. over night. The mixture was filtered, and a solution of NHC^Dipp^CuCl (117 mg, 0.240 mmol) in acetonitrile (2 mL) was added, instantly leading to the formation of a brown precipitate. The mixture was stirred for 15 min at r.t. to assure complete conversion of the reactants. After filtering off the supernatant solution (deep red) and removal of the solvent in vacuo, a brown solid was obtained. The solid was dissolved in toluene (3 mL) and filtered to remove KCl formed during the reaction. The toluene solution was concentrated to half of its original volume and placed in a freezer at −40 °C for crystallization, yielding red plate-shaped crystals of the product (74 mg, 30%), suitable for single crystal X-ray diffraction.

Route B:

A solution of K_2_[Ge_9_R_2_] (R: Si(TMS)_3_) (92 mg, 0.075 mmol) in acetonitrile (1 mL) was treated with an acetonitrile solution (1.5 mL) of NHC^Dipp^CuCl (73 mg, 0.150 mmol), instantly leading to the formation of a brownish precipitate. The mixture was stirred for 15 min at r.t. to assure complete conversion of the reactants. Subsequently, the supernatant solution (slightly red) was filtered off, and the residue was dried in vacuo. The residue was dissolved in toluene (2 mL) and filtered to remove KCl formed during the reaction, and then the solution was concentrated to half of its original volume. The sample was stored in a freezer at -40 °C for recrystallization, yielding red crystals (69 mg, 45%) of the product.

**^1^H-NMR (400 MHz, 298 K, thf-*d*_8_):** δ[ppm] = 7.47–7.42 (m, 4H, CH_Ph(p)_), 7.32 (d, *^3^J*_HH_ = 7.8 Hz, 8H, CH_Ph(m)_), 7.23 (s, 4H, CH_Im_), 2.77 (hept, *^3^J*_HH_ = 6.8 Hz, 8H, CH*_i_*_Pr_), 1.46 (d, *^3^J*_HH_ = 6.9 Hz, 24H, Me*_i_*_Pr_), 1.09 (d, *^3^J*_HH_ = 6.9 Hz, 24H, Me*_i_*_Pr_), 0.05 (s, 54H, Me_TMS_).

**^1^H-NMR (400 MHz, 298 K, C_6_D_6_):** δ[ppm] = 7.35–7.30 (m, 4H, CH_Ph(p)_), 7.23 (d, *^3^J*_HH_ = 7.8 Hz, 8H, CH_Ph(m)_), 6.22 (s, 4H, CH_Im_), 2.79 (hept, *^3^J*_HH_ = 6.8 Hz, 8H, CH*_i_*_Pr_), 1.63 (d, *^3^J*_HH_ = 6.9 Hz, 24H, Me*_i_*_Pr_), 1.01 (d, *^3^J*_HH_ = 6.9 Hz, 24H, Me*_i_*_Pr_), 0.41 (s, 54H, Me_TMS_).

**^13^C-NMR (101 MHz, 298 K, thf-*d*_8_):** δ[ppm] = 146.12 (s, C_Ph(*i*Pr)_), 136.68 (s, C_Ph_N), 130.83 (s, CH_Ph(p)_), 125.13 (s, CH_Ph(m)_), 124.01 (s, CH_Im_), 29.51 (s, CH*_i_*_Pr_), 25.83 (s, Me*_i_*_Pr_), 3.40 (s, Me_TMS_).

**^29^Si-INEPT NMR: (79 MHz, 298 K, thf-*d*_8_):** δ[ppm] = −10.63 (s, Si_TMS_), −105.36 (s, Si_Ge9_).

**ESI-MS**: *m*/*z* 1600.8 [NHC^Dipp^Cu{Ge_9_(Si(TMS)_3_)_2_}]^−^, 2092.8 [(NHC^Dipp^Cu)_2_{Ge_9_(Si(TMS)_3_)_2_}K]^+^.

**Elemental analysis:** Anal. Calcd. for C_72_H_128_Ge_9_Cu_2_N_4_Si_8_: C, 42.1; H, 6.2; N, 2.7; found: C, 42.0; H, 6.3; N, 2.7.

#### (NHC^Dipp^Ag)_2_{η^3^-Ge_9_(Si(TMS)_3_)_2_} (**3**)

Route A:

K_12_Ge_17_ (204.5 mg, 0.120 mmol) was treated with Si(TMS)_3_Cl (204.0 mg, 0.720 mmol) in a heterogeneous reaction in acetonitrile (2 mL) to form a deep red solution upon stirring at r.t. over night. The mixture was filtered, and a solution of NHC^Dipp^AgCl (128 mg, 0.240 mmol) in acetonitrile (2 mL) was added, instantly leading to the formation of a brown precipitate. Filtering off the supernatant solution (deep red) and removal of the solvent in vacuo led to a brown solid which was dissolved in toluene (3 mL) and filtered to remove KCl formed during the reaction. The toluene solution was concentrated to half of its original volume and placed in a freezer at −40 °C for crystallization. However, crystals suitable for single crystal X-ray diffraction could not be obtained yet. The crude product was obtained as a brownish solid after removal of toluene (51 mg, 20%).

Route B:

A solution of K_2_[Ge_9_R_2_] (R: Si(TMS)_3_) (92 mg, 0.075 mmol) in acetonitrile (1 mL) was treated with an acetonitrile solution (1.5 mL) of NHC^Dipp^AgCl (80 mg, 0.150 mmol), instantly leading to the formation of a brownish precipitate. The mixture was stirred for 15 min at r.t. to assure complete conversion of the reactants. The supernatant solution (slightly red) was filtered off, and the residue was dried in vacuo. The residue was dissolved in toluene (2 mL) and filtered to remove KCl formed during the reaction, and the solution was concentrated to half of its original volume. The sample was stored in a freezer at −40 °C. However, crystals suitable for single crystal X-ray diffraction could not be obtained yet. The crude product was obtained as a brownish solid after removal of toluene (40 mg, 25%).

**^1^H-NMR (400 MHz, 298 K, thf-*d*_8_):** δ[ppm] = 7.46–7.42 (m, 4H, CH_Ph(p)_), 7.40 (s, 4H, CH_Im_), 7.30 (d, *^3^J*_HH_ = 7.8 Hz, 8H, CH_Ph(m)_), 2.70 (hept, *^3^J*_HH_ = 6.8 Hz, 4H, CH*_i_*_Pr_), 1.43 (d, *^3^J*_HH_ = 6.9 Hz, 24H, Me*_i_*_Pr_), 1.14 (d, *^3^J*_HH_ = 6.9 Hz, 24H, Me*_i_*_Pr_), 0.01 (s, 54H, Me_TMS_).

**^13^C-NMR (101 MHz, 298 K, thf-*d*_8_):** δ[ppm] = 146.31 (s, C_Ph(*i*Pr)_), 136.56 (s, C_Ph_N), 130.97 (s, CH_Ph(p)_), 125.04 (s, CH_Ph(m)_), 124.29 (s, CH_Im_), 29.56 (s, CH*_i_*_Pr_), 26.28 (s, Me*_i_*_Pr_), 24.67 (s, Me*_i_*_Pr_), 3.36 (s, Me_TMS_).

**^29^Si-INEPT NMR: (79 MHz, 298 K, thf-*d_8_*):** δ[ppm] = −10.38 (s, Si_TMS_), −105,75 (s, Si_Ge9_).

**ESI-MS**: *m/z* 1644.8 [NHC^Dipp^Ag{Ge_9_(Si(TMS)_3_)_2_}]^−^.

#### (NHC^Dipp^Au)_2_{η^3^-Ge_9_(Si(TMS)_3_)_2_} (**4**)

Route A:

K_12_Ge_17_ (204.5 mg, 0.120 mmol) was treated with Si(TMS)_3_Cl (204.0 mg, 0.720 mmol) in a heterogeneous reaction in acetonitrile (2 mL) to form a deep red solution upon stirring at r.t. over night. The mixture was filtered, and a solution of NHC^Dipp^AuCl (149 mg, 0.240 mmol) in acetonitrile (3 mL) was added, instantly leading to the formation of a brown precipitate. Filtering off the supernatant solution (deep red), and removal of the solvent in vacuo led to a brown solid which was dissolved in toluene (3 mL) and filtered to remove KCl formed during the reaction. The toluene solution was concentrated to half of its original volume and placed in a freezer at −40 °C for crystallization. However, crystals suitable for single crystal X-ray diffraction could not be obtained yet. The crude product was obtained as a brownish solid after removal of toluene (83 mg, 30%).

Route B:

A solution of K_2_[Ge_9_R_2_] (R: Si(TMS)_3_) (92 mg, 0.075 mmol) in acetonitrile (1 mL) was treated with an acetonitrile solution (2 mL) of NHC^Dipp^AuCl (93 mg, 0.150 mmol), instantly leading to the formation of a brownish precipitate. The mixture was stirred for 15 min at r.t. to assure complete conversion of the reactants. The supernatant solution (light red) was filtered off, and the residue was dried in vacuo. The residue was dissolved in toluene (2 mL) and filtered to remove KCl formed during the reaction, and the solution was concentrated to half of its original volume. The sample was stored in a freezer at −40 °C. However, crystals suitable for single crystal X-ray diffraction could not be obtained yet. The crude product was obtained as a brownish solid after removal of toluene (52 mg, 30%).

**^1^H-NMR (400 MHz, 298 K, C_6_D_6_):** δ[ppm] = 7.32–7.28 (m, 4H, CH_Ph(p)_), 7.20 (d, *^3^J*_HH_ = 7.7 Hz, 8H, CH_Ph(m)_), 6.28 (s, 4H, CH_Im_), 2.73 (hept, *^3^J*_HH_ = 6.6 Hz, 8H, CH*_i_*_Pr_), 1.61 (d, *^3^J*_HH_ = 6.9 Hz, 24H, Me*_i_*_Pr_), 1.04 (d, *^3^J*_HH_ = 6.9 Hz, 24H, Me*_i_*_Pr_), 0.43 (s, 54H, Me_TMS_).

**^1^H-NMR (400 MHz, 298 K, thf-*d*_8_):** δ[ppm] = 7.45-7.38 (m, 8H, CH_Ph(p)_ + CH_Im_), 7.27 (d, *^3^J*_HH_ = 7.7 Hz, 8H, CH_Ph(m)_), 2.74 (hept, *^3^J*_HH_ = 6.6 Hz, 8H, CH*_i_*_Pr_), 1.43 (d, *^3^J*_HH_ = 6.9 Hz, 24H, Me*_i_*_Pr_), 1.13 (d, *^3^J_HH_* = 6.9 Hz, 24H, Me*_i_*_Pr_), 0.09 (s, 54H, Me_TMS_).

**^13^C-NMR (101 MHz, 298 K, C_6_D_6_):** δ[ppm] = 145.43 (s, C_Ph(*i*Pr)_), 135.16 (s, C_Ph_N), 130.84 (s, CH_Ph(p)_), 124.75 (s, CH_Ph(m)_), 122.38 (s, CH_Im_), 28.92 (s, CH*_i_*_Pr_), 25.52 (s, Me*_i_*_Pr_), 24.63 (s, Me*_i_*_Pr_), 3.45 (s, Me_TMS_).

**^29^Si-INEPT NMR (79 MHz, 298 K, C_6_D_6_):** δ[ppm] = −9.72 (s, Si_TMS_), −105.49 (s, Si_Ge9_).

**ESI-MS**: *m/z* 1734.8 [NHC^Dipp^Au{Ge_9_(Si(TMS)_3_)_2_}]^−^, 2359.0 [(NHC^Dipp^Au)_2_{Ge_9_(Si(TMS)_3_)_2_}K]^+^.

#### (NHC^Mes^Cu)_2_{η^3^-Ge_9_(Si(TMS)_3_)_2_} (**5**)

A solution of K_2_[Ge_9_R_2_] (R: Si(TMS)_3_) (92 mg, 0.075 mmol) in acetonitrile (1 mL) was treated with an acetonitrile solution (2 mL) of NHC^Mes^CuCl (60.5 mg, 0.150 mmol), instantly leading to the formation of a brownish precipitate. The mixture was stirred for 15 min at r.t. to assure complete conversion of the reactants. The supernatant solution (light red) was filtered off, and the residue was dried in vacuo. The residue was dissolved in toluene (2 mL) and filtered to remove KCl formed during the reaction. The sample was stored in a freezer at −40 °C. However, crystals suitable for single crystal X-ray diffraction could not be obtained yet. The crude product was obtained as a brownish solid after removal of toluene (59 mg, 40%).

**^1^H-NMR (400 MHz, 298 K, thf-*d*_8_):** δ[ppm] = 7.13 (s, 4H, CH_Im_), 7.01 (s, 8H, CH_Ph_), 2.42 (s, 12H, Me_p_), 2.10 (s, 24H, Me_o_), 0.08 (s, 54H, Me_TMS_).

**^13^C-NMR (101 MHz, 298 K, thf-*d*_8_):** δ[ppm] = 139.35 (s, C_Ph(Me(p))_), 136.57 (s, C_Ph_N), 135.69 (s, C_Ph(Me(o))_) 130.54 (s, CH_Ph_), 121.98 (s, CH_Im_), 21.72 (s, CH_Me(p)_), 18.69 (s, Me _Me(o)_), 3.18 (s, Me_TMS_).

**^29^Si-INEPT NMR (79 MHz, 298 K, thf-*d*_8_):** δ[ppm] = −10.24 (s, Si_TMS_), −108.84 (s, Si_Ge9_).

**ESI-MS**: *m*/*z* 1516.6 [NHC^Mes^Cu{Ge_9_(Si(TMS)_3_)_2_}]^−^.

## 4. Conclusions

Within this work, we studied the silylation reaction of K_12_Ge_17_ and the reactivity of bis- and tris-silylated [Ge_9_] clusters towards coinage metal carbene complexes NHC^Dipp^*M*Cl (*M*: Cu, Ag, Au). The reaction of K_12_Ge_17_ with 6 eq. of Si(TMS)_3_Cl yielded the bis-silylated cluster [Ge_9_{Si(TMS)_3_}_2_]^2−^ as the main product in solution, contrasting the analogue reaction of K_4_Ge_9_, which exclusively results in the formation of [Ge_9_{Si(TMS)_3_}_3_]^−^. The subsequent reaction of the obtained solutions, as well as the reaction of pure [Ge_9_{Si(TMS)_3_}_2_]^2−^ with NHC^Dipp^*M*Cl, yielded the novel neutral dinuclear *Zintl* cluster coinage metal NHC compounds (NHC*M*)_2_{η^3^-Ge_9_R^I^_2_} (*M* = Cu, Ag, Au; NHC = NHC^Dipp^, NHC^Mes^ and R^I^ = Si(TMS)_3_) (**2**–**5**). Furthermore, the reaction of [Ge_9_{Si(*^i^*Pr)_3_}_3_]^−^ with NHC^Dipp^CuCl gave the neutral compound NHC^Dipp^Cu{Ge_9_R_3_} (R = Si(*^i^*Pr)_3_) (**1**), which showed different structural features compared to its analogues bearing the larger silyl groups [Si(*^i^*Bu)_3_]^+^ and [Si(TMS)_3_]^+^.

## Figures and Tables

**Table 1:** Selected distances and angles in compound **1** and its analogues NHC^Dipp^Cu{η^3^-Ge_9_R_3_} (R = Si(*^i^*Bu)_3_, Si(TMS)_3_).

Distances [Å]	1	R = Si(*^i^*Bu)_3_ [19]	R = Si(TMS)_3_ [24]
*d*_1_(Cu-Ge1)	2.4914(8)	2.4911(6)	2.4943(9)
*d*_2_(Cu-Ge2)	2.541(1)	2.4933(6)	2.5188(9)
*d*_3_(Cu-Ge3)	2.5661(8)	2.5594(6)	2.5660(9)
*d*_mean_(Cu-Ge) ^a^	2.5328(9)	2.5146(6)	2.5263(9)
*d*(Cu-C1) ^b^	1.951(3)	1.943(3)	1.957(5)
angles [deg.]			
ctp-Cu-C1 ^c^	177.67(4)	172.77(2)	173.59(1)

^a^ The average Cu-Ge distances are given as *d*_mean_(Cu-Ge). ^b^ Distances between Cu^+^ and carbene carbon atoms are given as *d*(Cu-C1). ^c^ ctp: center of gravity of the coordinating atoms of the triangular face of the [Ge_9_] cluster.

**Table 2:** Selected distances and angles in compound **2**.

Distances [Å]	2	
	A	B
*d*_1_(Cu-Ge)	2.601(1)	2.532(1)
*d*_2_(Cu-Ge)	2.532(1)	2.582(1)
*d*_3_(Cu-Ge)	2.405(1)	2.413(1)
*d*_mean_(Cu-Ge) ^a^	2.513(1)	2.509(1)
*d*(Cu-C_carbene_)	1.941(5)	1.913(5)
angles [deg.]		
ctp-Cu-C_carbene_^b^	162.88(2)	163.92(2)

The copper-germanium distances *d*(Cu-Ge) are defined as follows: **A**: *d*_1_(Cu1-Ge2), *d*2(Cu1-Ge5), *d*3(Cu1-Ge9); **B:** *d*1(Cu2-Ge3), *d*2(Cu2-Ge4), *d*3(Cu2-Ge7). Labelling is given in in Figure 2. ^a^ The average Cu-Ge distances are given as *d*_mean_(Cu-Ge). ^b^ ctp: center of gravity of the coordinating atoms of the triangular faces of the [Ge_9_] cluster.

**Table 3:** Crystallographic data for compounds **1** and **2**.

Compound	1	2
formula	C_54_H_99_Ge_9_Cu_1_N_2_Si_3_·2 tol	C_72_H_128_Ge_9_Cu_2_N_4_Si_8_·4 tol.
fw (g·mol^−1^)	1761.73	2423.42
space group (no.)	*P*1 (2)	*P*1 (2)
*a* (Å)	12.377(3)	15.102(2)
*b* (Å)	13.387(3)	16.831(3)
*c* (Å)	26.468(5)	25.659(5)
*α* (deg.)	77.69(3)	108.951(9)
*β* (deg.)	78.76(3)	101.847(8)
*γ* (deg.)	77.58(3)	90.837(8)
*V* (Å^3^)	4133.0(2)	6013.8(2)
Z	2	2
*T* (K)	150(2)	100(2)
*λ* (Å)	Mo-*Kα*	Mo-*Kα*
ρ_calcd_ (g·cm^−3^)	1.416	1.338
*μ* (mm^−1^)	3.553	2.680
collected reflections	62327	90837
independent reflections	14985	21064
R_int_/R_δ_	0.0263/0.0187	0.0914/0.0943
parameters/restraints	776/0	1273/366
*R*_1_ (*I* > 2 *σ*(*I*)/all data)	0.0330/0.0414	0.0468/0.0985
w*R*_2_ (*I* > 2 *σ*(*I*)/all data)	0.0801/0.0854	0.0953/0.1126
goodness of fit	1.084	0.998
max./min. diff. el. density (e·Å^−3^)	0.710/−0.757	0.827/–0.653
CCDC	1553930	1553929

## Supplementary Materials

The following material is available online, Tables SI1–SI3: Selected bond lengths and angles of compounds **1** and **2**, as well as comparison of cluster shapes of both compounds to previously reported similar compounds. Figure SI 1: Full ellipsoid pictures of compounds **1** and **2**. Figure SI 2–SI 20: NMR spectra of compounds **1**–**5**.
