# Supplementary material for: N-Heterocyclic Carbene Coinage Metal Complexes of the Germanium-Rich Metalloid Clusters [Ge9R3]− and [Ge9RI2]2− with R = Si(iPr)3 and RI = Si(TMS)3
Source: Molecules. 2017 Jul 19;22(7):1204. doi: 10.3390/molecules22071204 (PMC6152075; doi:10.3390/molecules22071204)
Supplement: Supplementary file 1 [file molecules-22-01204-s001.pdf]

Supporting Information

Coinage Metal NHC Compounds of Germanium-Rich Metalloid Clusters $[Ge_9R_3]^-$ and $[Ge_9R^I_2]^{2-}$ with $R = Si(^iPr)_3$ and $R^I = Si(TMS)_3$

Felix S. Geitner [1#], Michael A. Giebel [2#], Alexander Pöthig [3] and Thomas F. Fässler [2,*]

[1] Felix S. Geitner, WACKER Institute for Silicon Chemistry and Department of Chemistry, Technische Universität München, Lichtenbergstraße 4, 85747 Garching, Germany.

[2] Michael A. Giebel, Department of Chemistry, Technische Universität München, Lichtenbergstraße 4, 85747 Garching, Germany.

[2,*] Prof. Dr. T. F. Fässler, Department of Chemistry, Technische Universität München, Lichtenbergstraße 4, 85747 Garching, Germany.

[3] Dr. Alexander Pöthig, TUM Catalysis Research Center (CRC), Ernst-Otto-Fischer Straße 1, 85747 Garching, Germany.

[#] Authors contributed equally to this work

Content

Selected Distances and Angles 2

NMR data 5

Selected Distances and Angles

Table SI 1: Selected bond lengths and angles of compound **1**.

bond	distance [Å]
Ge1-Ge2	2.8393(9)
Ge1-Ge3	2.8797(9)
Ge1-Ge4	2.5065(9)
Ge1-Ge6	2.5059(8)
Ge2-Ge3	2.834(1)
Ge2-Ge4	2.5189(8)
Ge2-Ge5	2.5205(8)
Ge3-Ge5	2.5211(9)
Ge3-Ge6	2.5261(8)
Ge4-Ge7	2.5473(9)
Ge4-Ge8	2.5631(8)
Ge5-Ge8	2.5578(9)
Ge5-Ge9	2.5401(8)
Ge6-Ge7	2.5547(9)
Ge6-Ge9	2.5572(8)
Ge7-Ge8	2.6421(9)
Ge8-Ge9	2.651(1)
Ge1-Ge7	3.4392(9)
Ge2-Ge8	3.3338(8)
Ge3-Ge9	3.2900(8)
Ge4-Si1	2.383(1)
Ge5-Si2	2.406(1)
Ge6-Si3	2.383(1)
Cu1-Ge1	2.4914(8)
Cu1-Ge2	2.5407(9)
Cu1-Ge3	2.5661(8)
Cu1-C1	1.951(3)
**atoms**	**angle [°]**
C1-Cu1-ctp1	177.67(4)

Table SI 2: Selected bond lengths and angles of compound **2**.

bond	distance [Å]
Ge1-Ge2	2.5909(9)
Ge1-Ge3	2.595(1)
Ge1-Ge4	2.591(1)
Ge1-Ge5	2.5781(9)
Ge2-Ge3	2.900(1)
Ge2-Ge5	2.7804(9)
Ge2-Ge6	2.5774(9)
Ge2-Ge9	2.8269(9)
Ge3-Ge4	2.805(1)
Ge3-Ge6	2.5521(8)
Ge3-Ge7	2.8189(9)
Ge4-Ge5	2.906(1)
Ge4-Ge7	2.8333(9)
Ge4-Ge8	2.5651(8)
Ge5-Ge8	2.5638(9)
Ge5-Ge9	2.8102(9)
Ge6-Ge7	2.5132(8)
Ge6-Ge9	2.5047(9)
Ge7-Ge8	2.5147(9)
Ge8-Ge9	2.505(1)
Ge2-Ge3	2.9003(3)
Ge4-Ge5	2.9054(3)
Ge7-Ge9	3.6969(4)
Ge6-Si1	2.388(2)
Ge8-Si5	2.43(1)
Cu1-Ge2	2.601(1)
Cu1-Ge5	2.532(1)
Cu1-Ge9	2.405(1)
Cu2-Ge3	2.532(1)
Cu2-Ge4	2.582(1)
Cu2-Ge7	2.413(1)
Cu1-C1	1.941(5)
Cu2-C4	1.913(5)
**atoms**	**angle [°]**
C1-Cu1-ctp1	162.88(2)
C4-Cu2-ctp2	163.93(2)

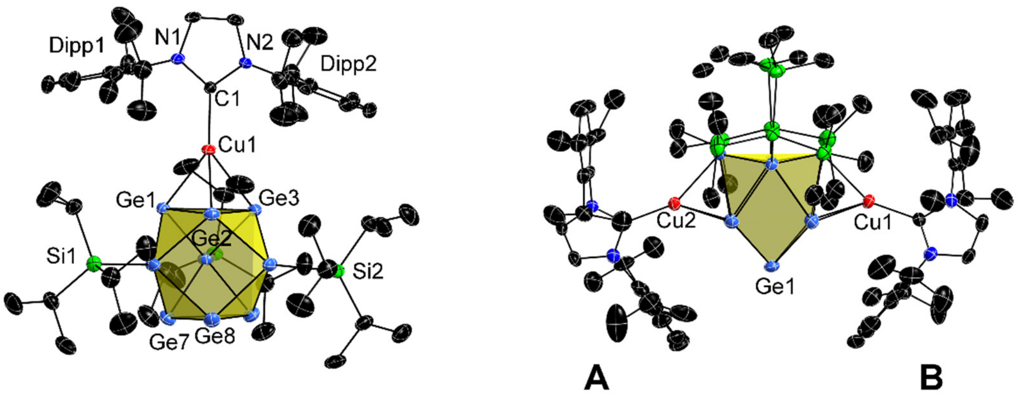

Figure SI 1. Molecular structure of compound **1** (left) and compound **2** (right). Displacement ellipsoids are shown at a 50 % probability level. For clarity, hydrogen atoms and co-crystallized toluene molecules are omitted. For **2**, the two [NHC^{Dipp}Cu]$^+$ moieties are labelled as **A** and **B**. Selected bond lengths and angles are summarized in Table SI 1 (**1**) and Table SI 2 (**2**).

Table SI 3: Comparison of the *Zintl* cluster shape in compounds **1**, **2** and [NHC^{Dipp}Cu{η^3-Ge$_9$R$_3$}] (R = Si(^{i}Bu)$_3$, Si(TMS)$_3$).

distances [Å]	**1**	**2**	R = Si(^{i}Bu)$_3$	R = Si(TMS)$_3$
h_1	3.4392(9)	2.9003(3)	3.4235(1)	3.3253(8)
h_2	3.3338(8)	2.9054(3)	3.3918(1)	3.3029(8)
h_3	3.2900(8)	3.6969(4)	3.1947(1)	3.4028(8)
maximum difference	0.1492(9)	0.7966(3)	0.2288(1)	0.0999(8)

Heights of the trigonal prism in compound **2** are defined as: h1 (Ge2-Ge3), h2 (Ge4-Ge5) and h3 (Ge7-Ge9). For all other compounds heights are defined as: h1 (Ge1-Ge7), h2 (Ge2-Ge8) h3 (Ge3-Ge9).

In *Zintl* cluster coinage metal NHC compounds, the contained silylated *Zintl* cluster ligands reveal either D_{3h}- or C_{2v}-symmetry. Assuming D_{3h}-symmetry the clusters can be described as tricapped trigonal prisms with the capping atoms bearing the silyl groups. In case of perfect D_{3h}-symmetry the heights of the trigonal prism (h) would all be equal. Regarding the data summarized in Table SI 3 it becomes obvious, that the clusters reveal most perfect D_{3h}-symmetry in NHC^{Dipp}Cu{η^3-Ge$_9$(Si(TMS)$_3$)$_3$} (smallest difference between minimum and maximum height of the trigonal prism). In compound **1** and NHC^{Dipp}Cu{η^3-Ge$_9$(Si(^{i}Bu)$_3$)$_3$} the trigonal prisms within the clusters are slightly more distorted. By contrast for novel compound **2** one of the heights is significantly longer than the others, which manifests the C_{2v}-symmetry of the [Ge$_9$] cluster in **2**.

NMR data

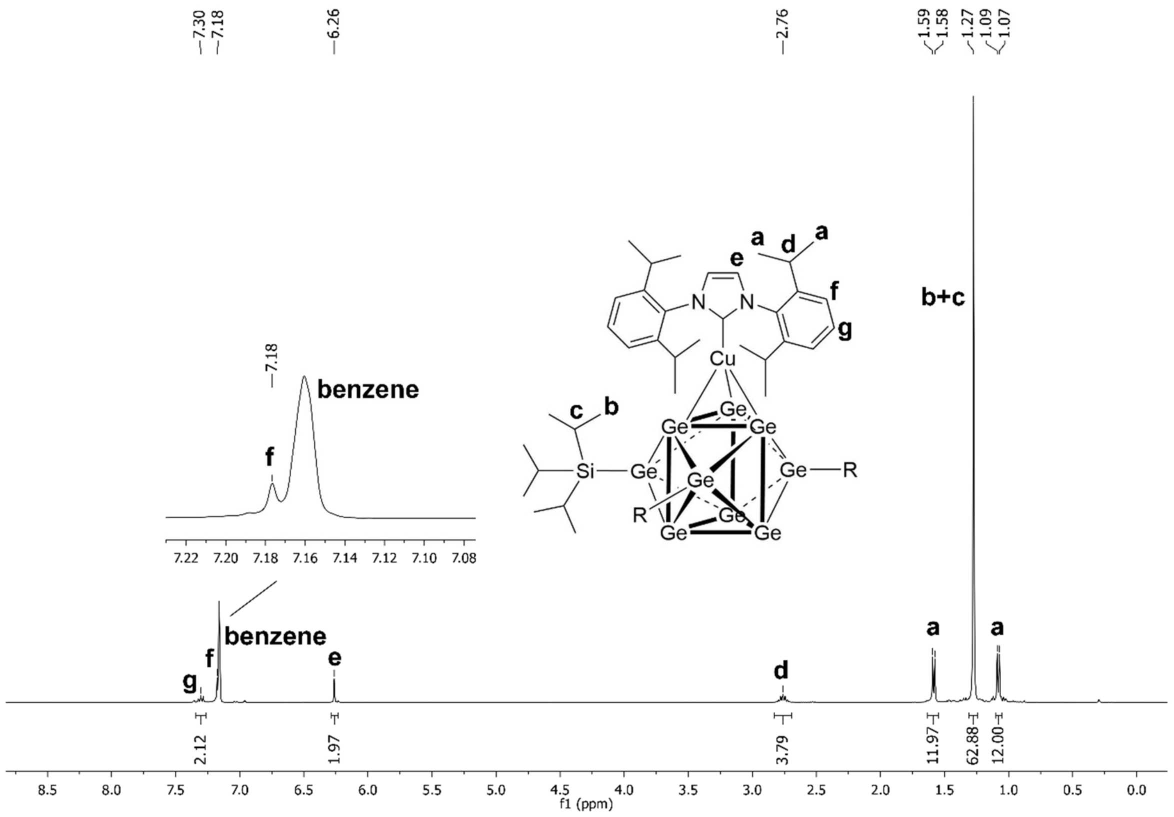

Figure SI 2: ^{1}H NMR of compound **1** in C_6D_6.

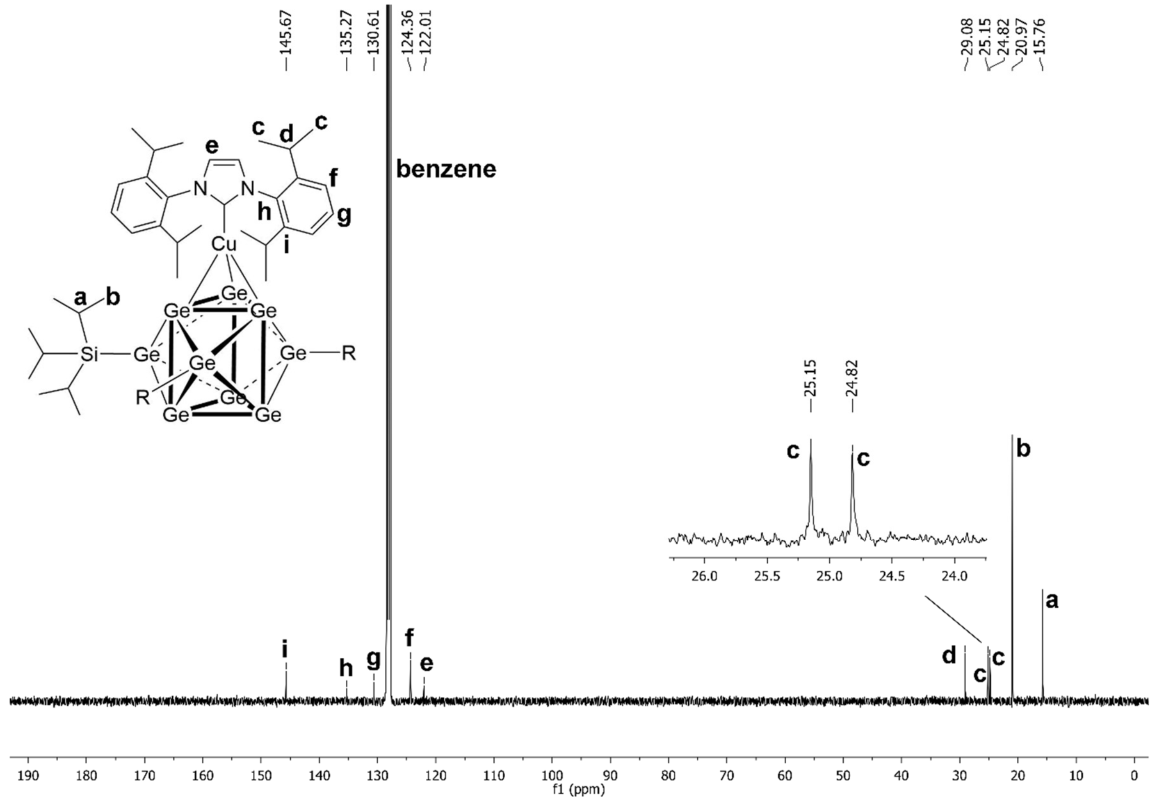

Figure SI 3: ^{13}C NMR of compound **1** in C_6D_6.

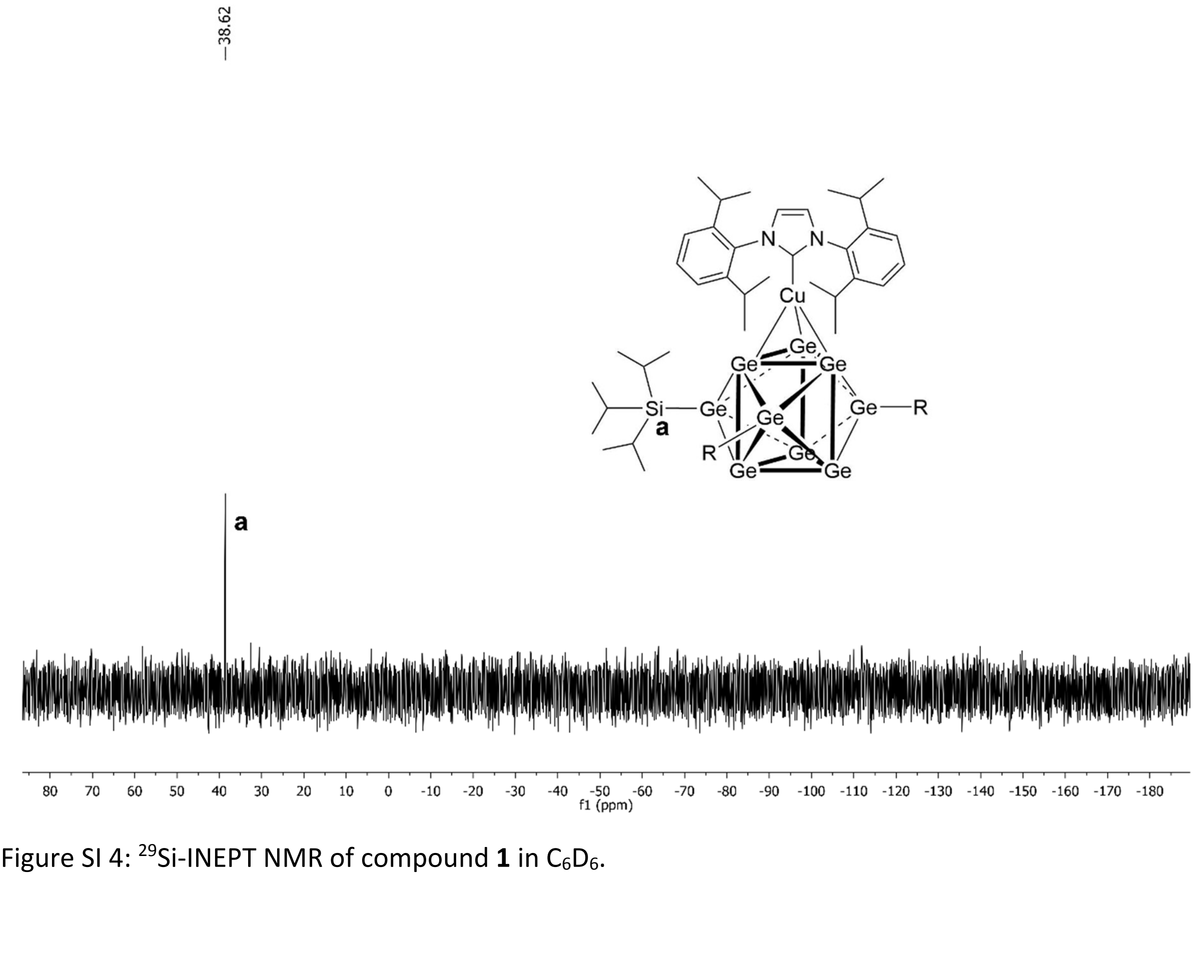

Figure SI 4: ^{29}Si-INEPT NMR of compound **1** in C_6D_6.

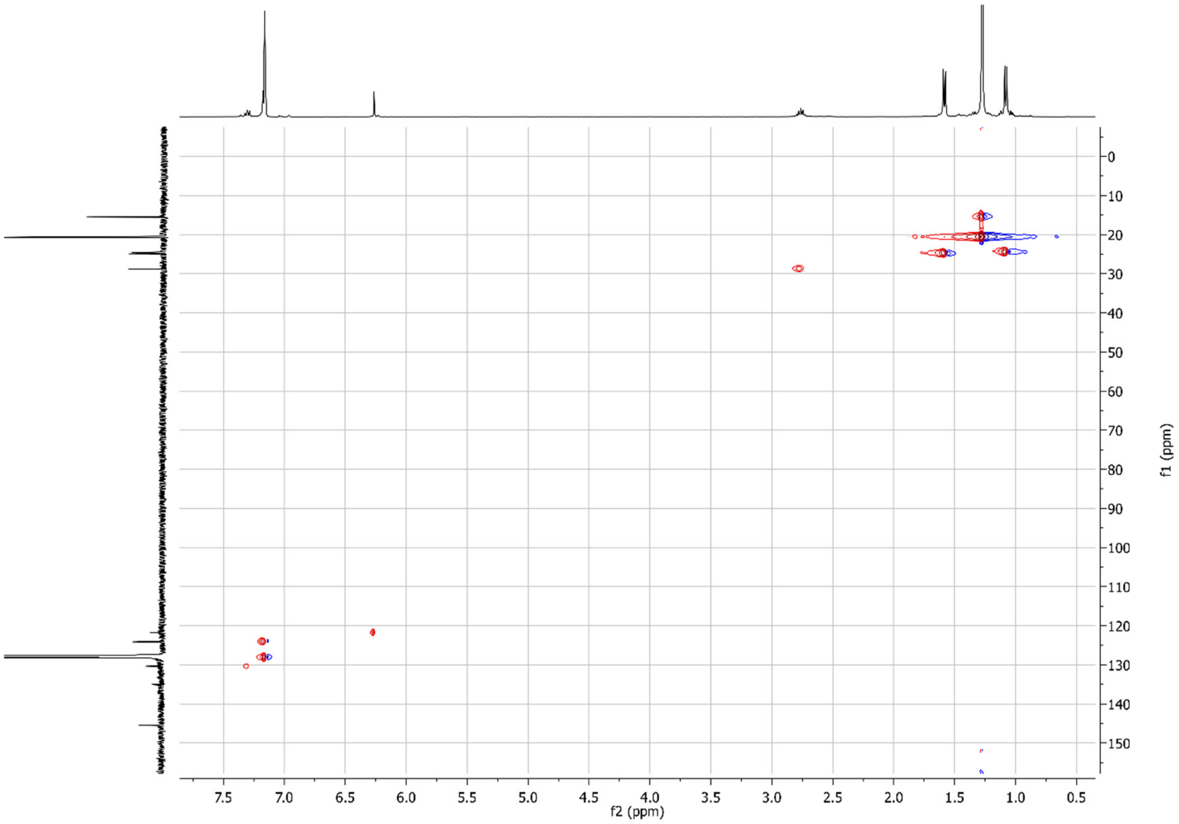

Figure SI 5: 2D-HSQC NMR of compound **1** in C_6D_6 revealing $Me_{iPr(silyl)}$ and $CH_{iPr(silyl)}$ to appear as one signal (pseudo singlet) in the ^1H NMR spectrum.

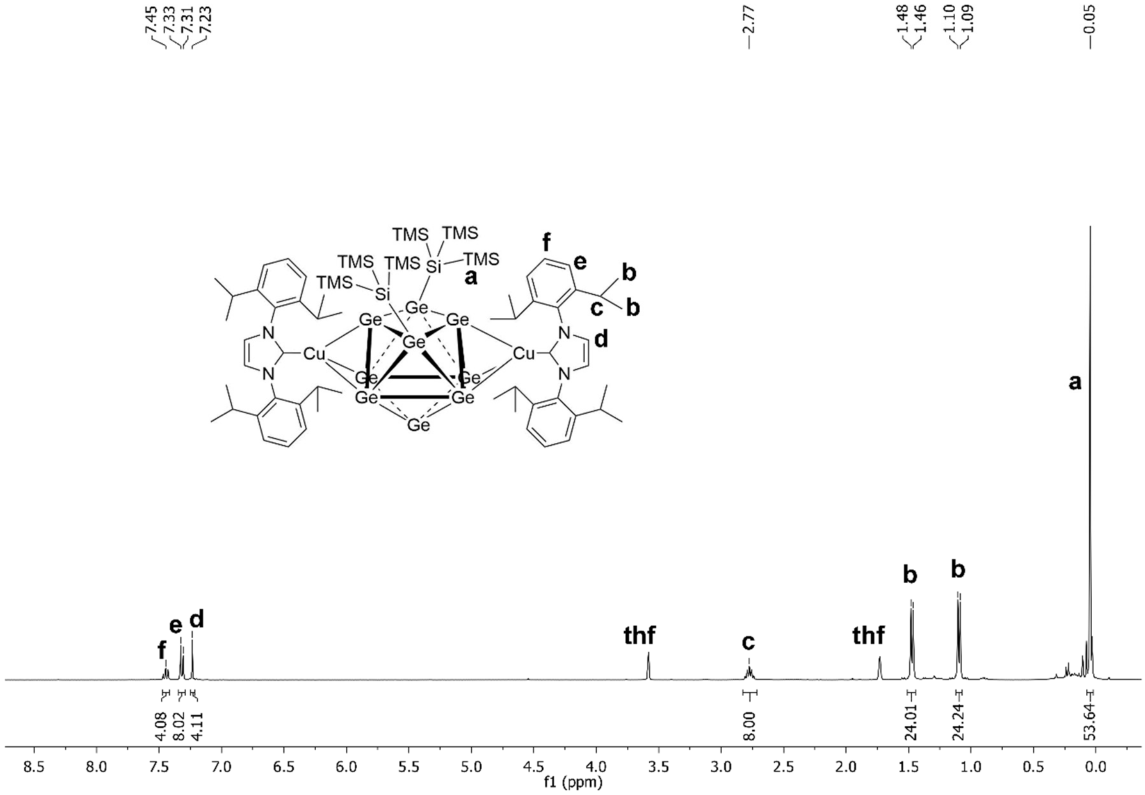

Figure SI 6: ^1H NMR of compound **2** in thf-d_8.

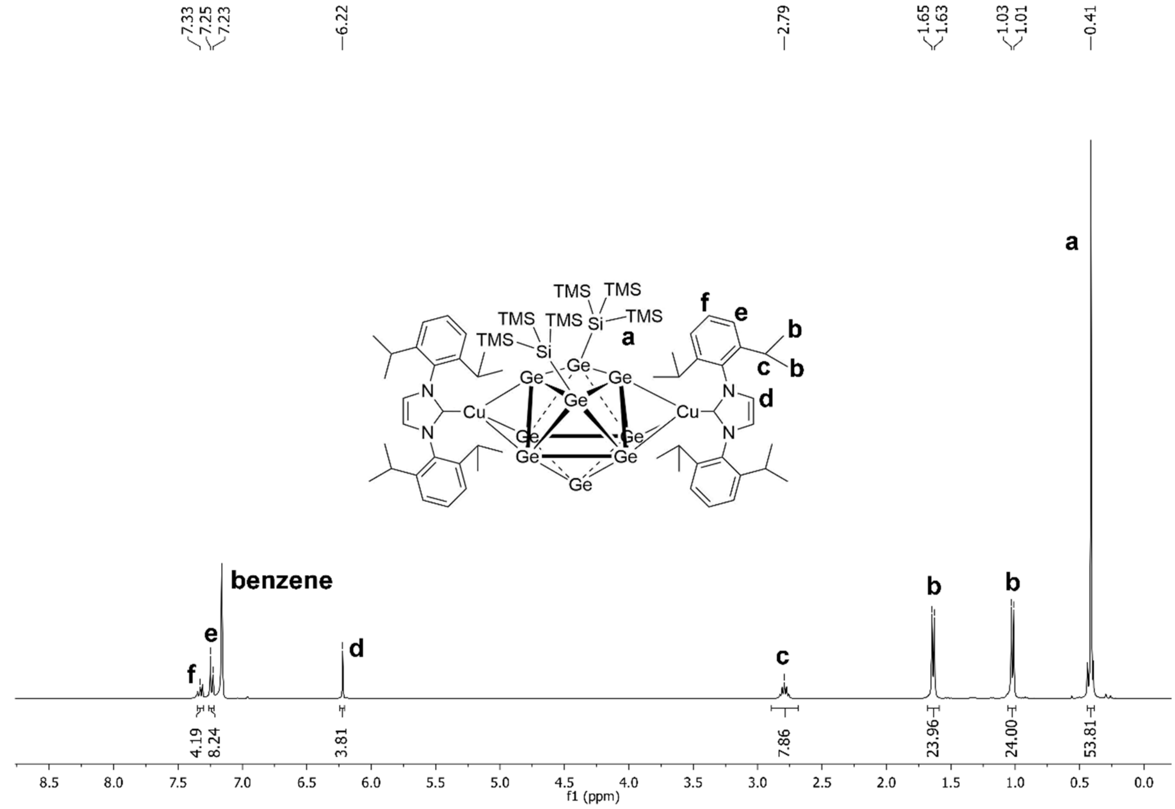

Figure SI 7: ^{1}H NMR of compound **2** in C_6D_6.

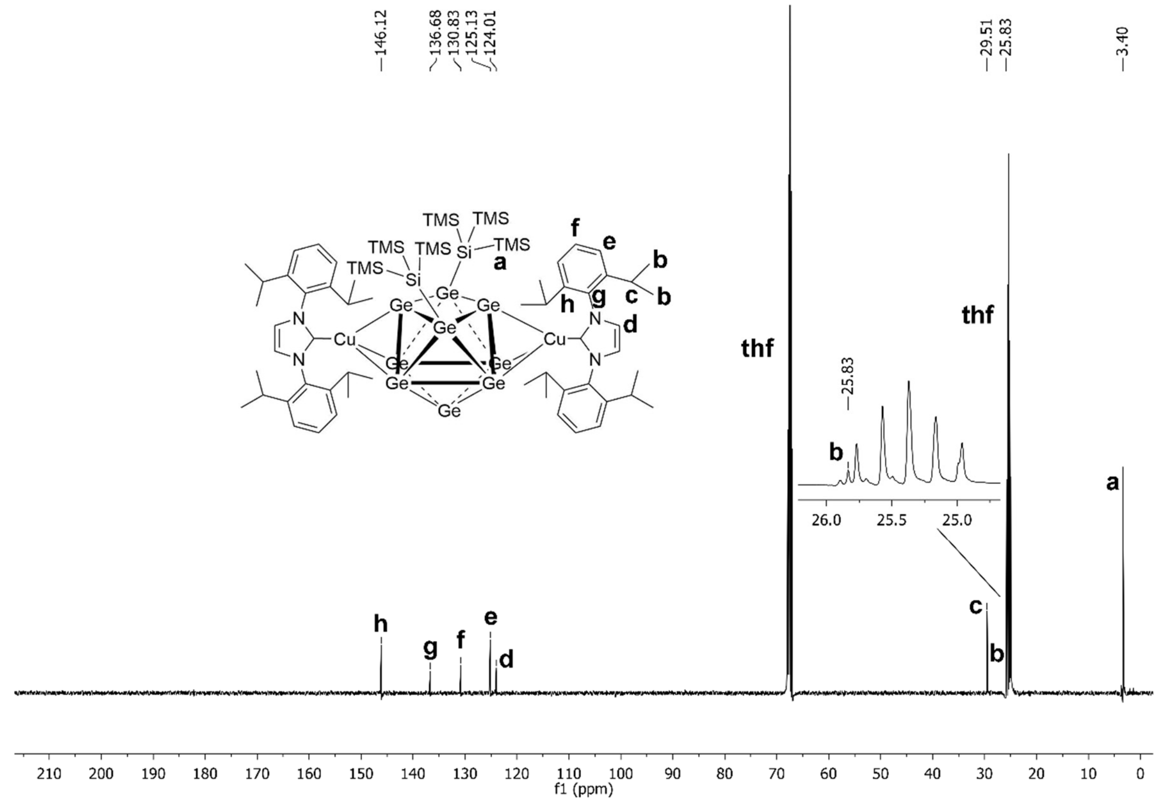

Figure SI 8: ^{13}C NMR of compound **2** in thf-d_8.

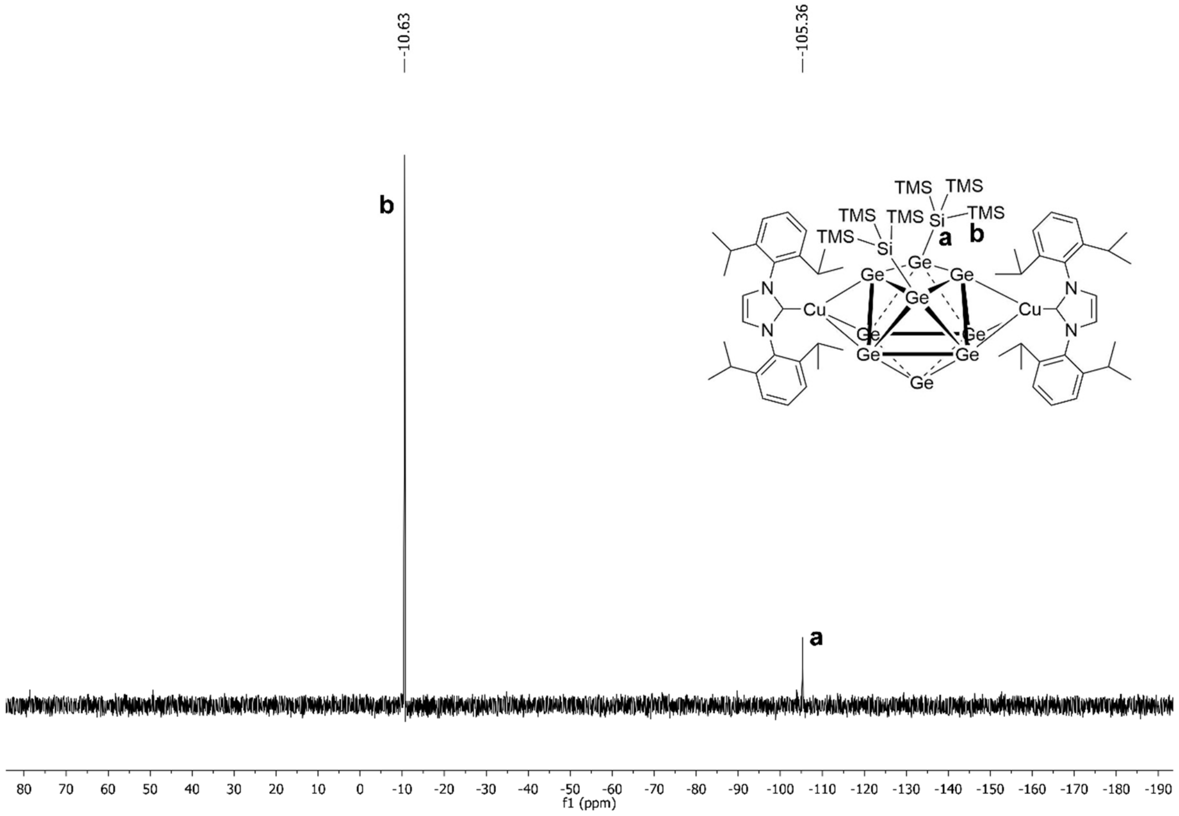

Figure SI 9: ^{29}Si-INEPT NMR of compound **2** in thf-d_8.

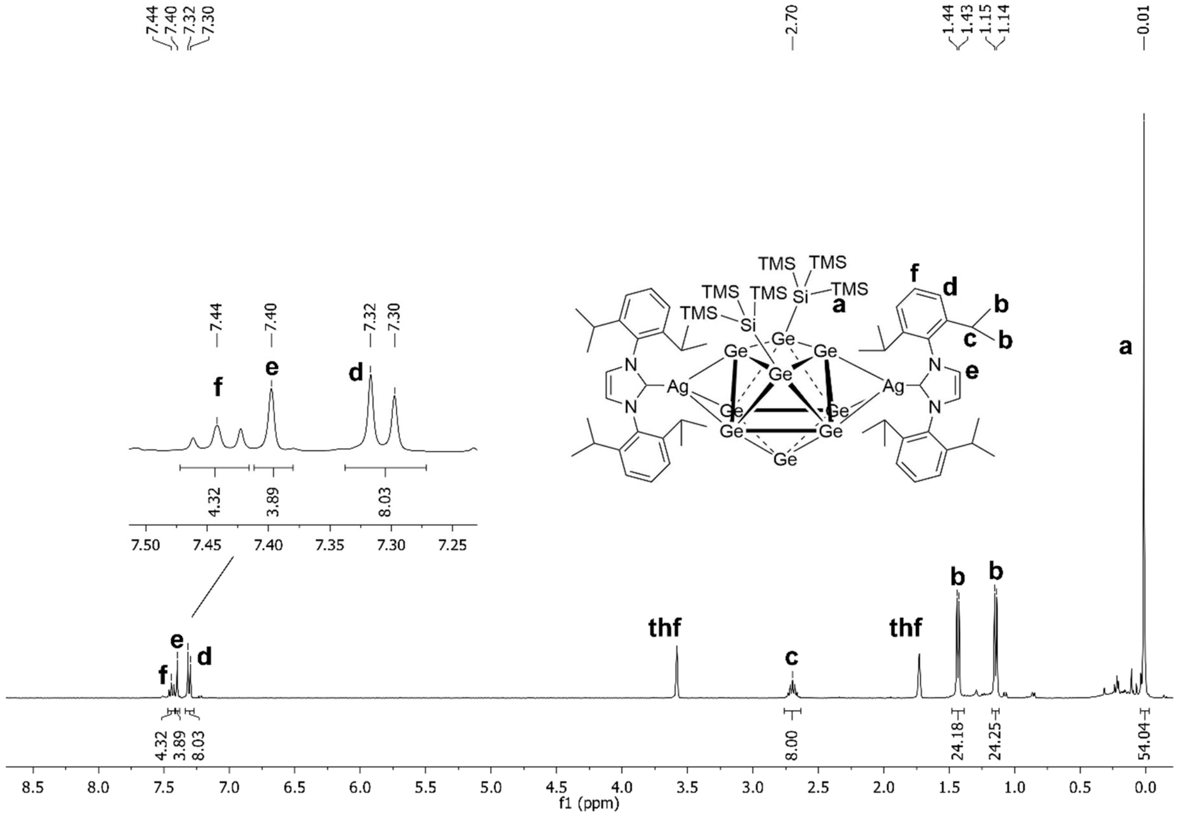

Figure SI 10: ^{1}H NMR of compound **3** in thf-d_8.

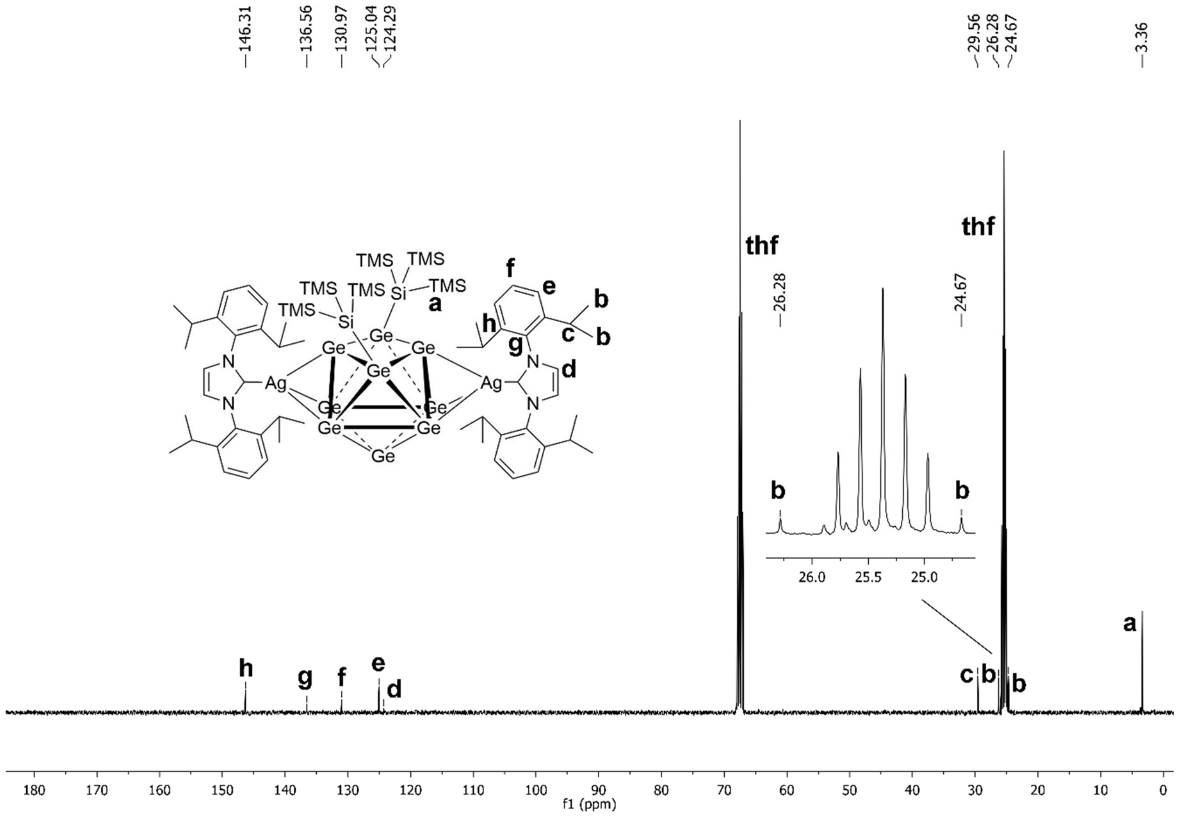

Figure SI 11: ^{13}C NMR of compound **3** in thf-d_8.

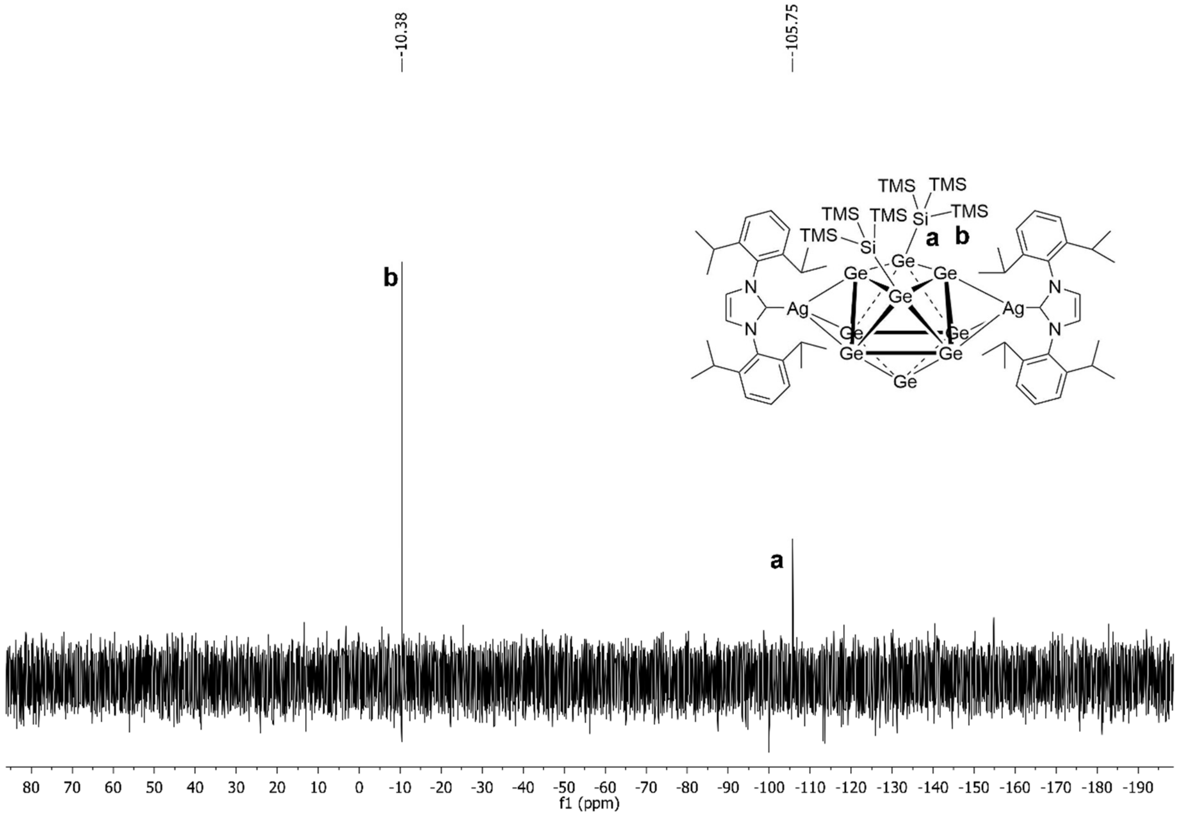

Figure SI 12: ^{29}Si-INEPT NMR of compound **3** in thf-d_8.

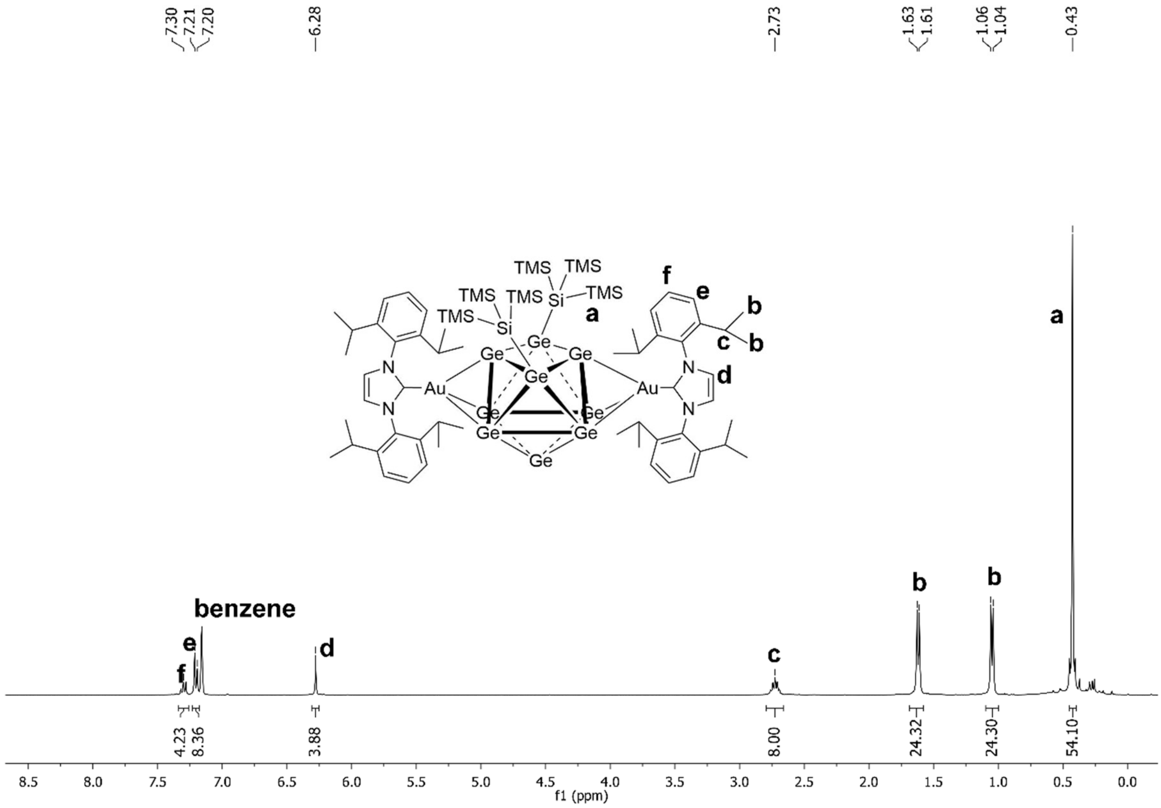

Figure SI 13: ^{1}H NMR of compound **4** in C_6D_6.

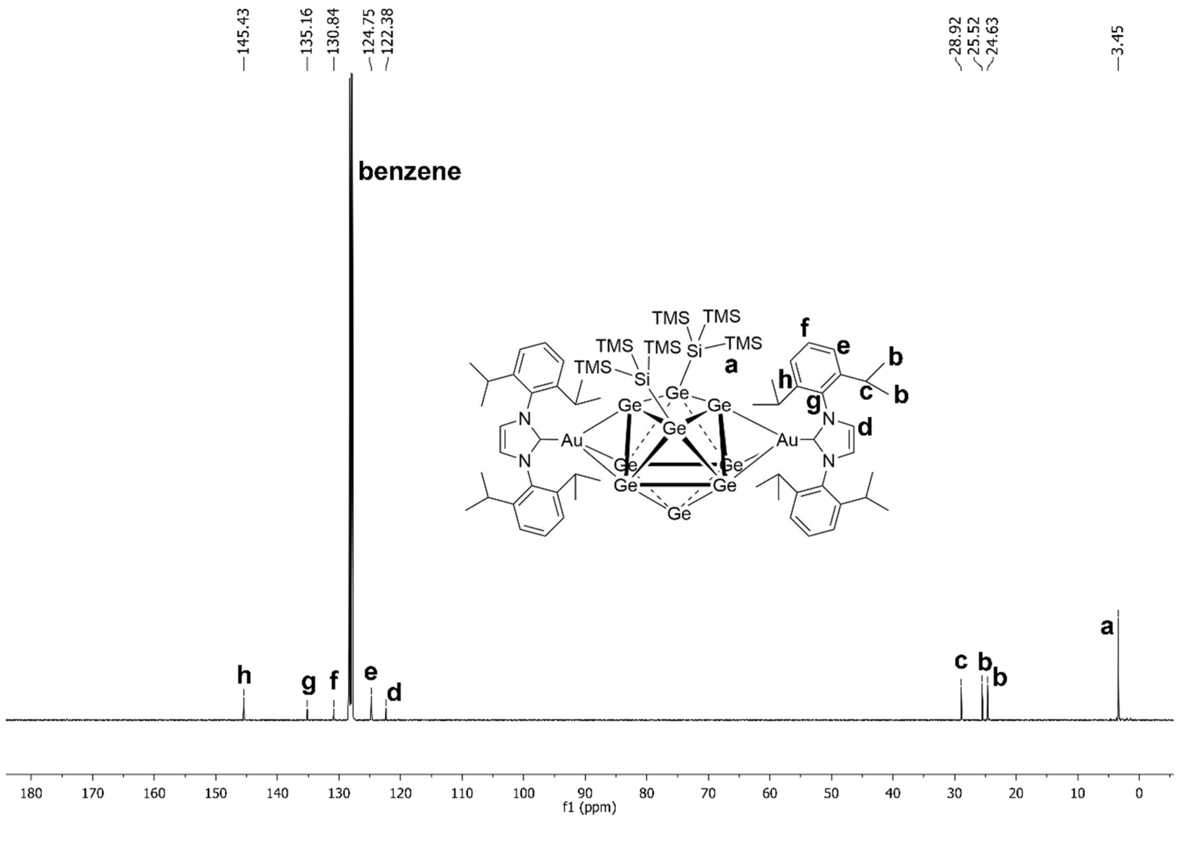

Figure SI 14: ^{13}C NMR of compound **4** in C_6D_6.

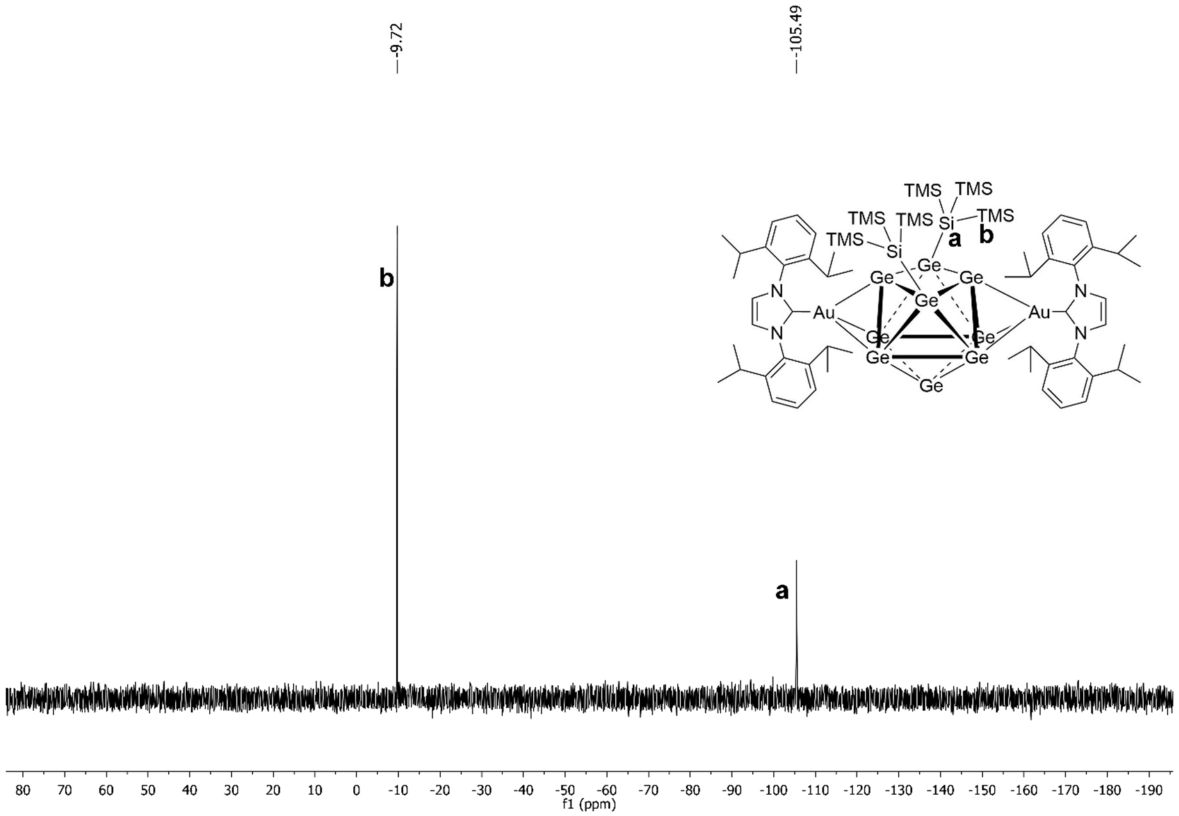

Figure SI 15: ^{29}Si-INEPT NMR of compound **4** in C_6D_6.

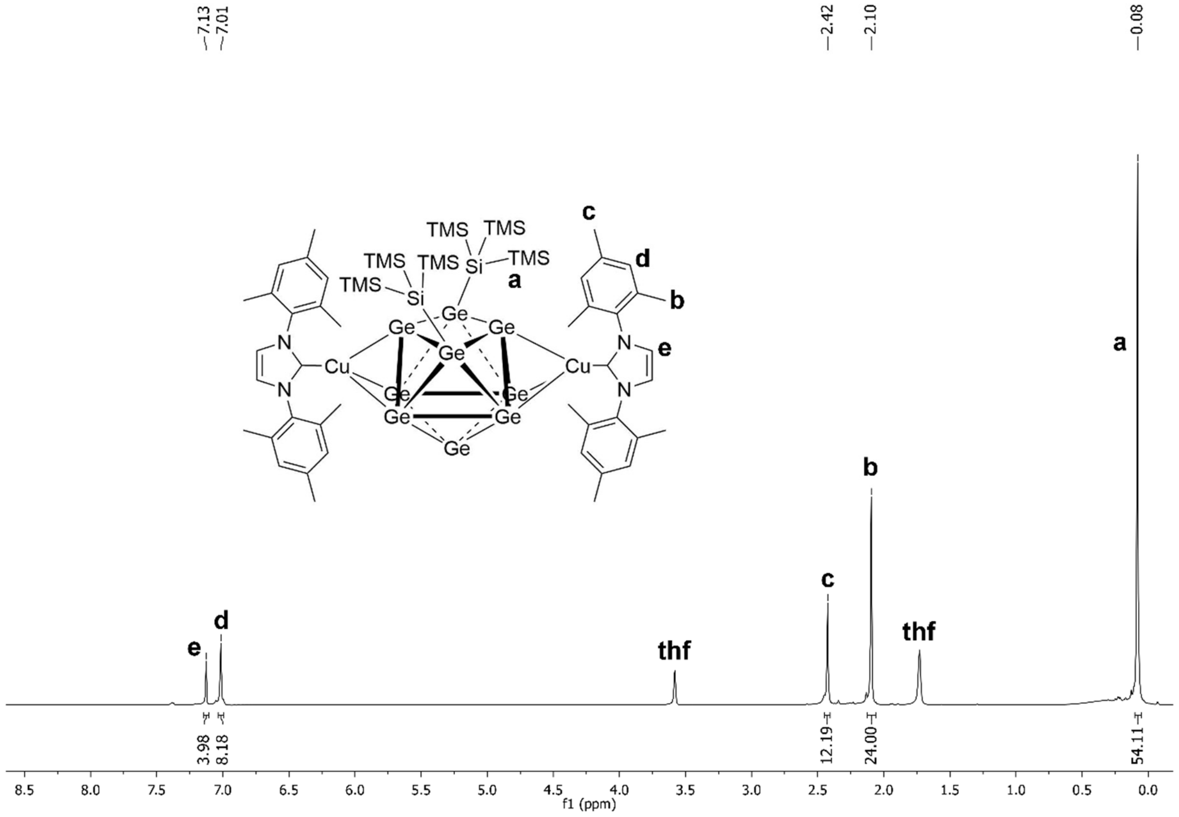

Figure SI 16: ^{1}H NMR of compound **5** in thf-d_8.

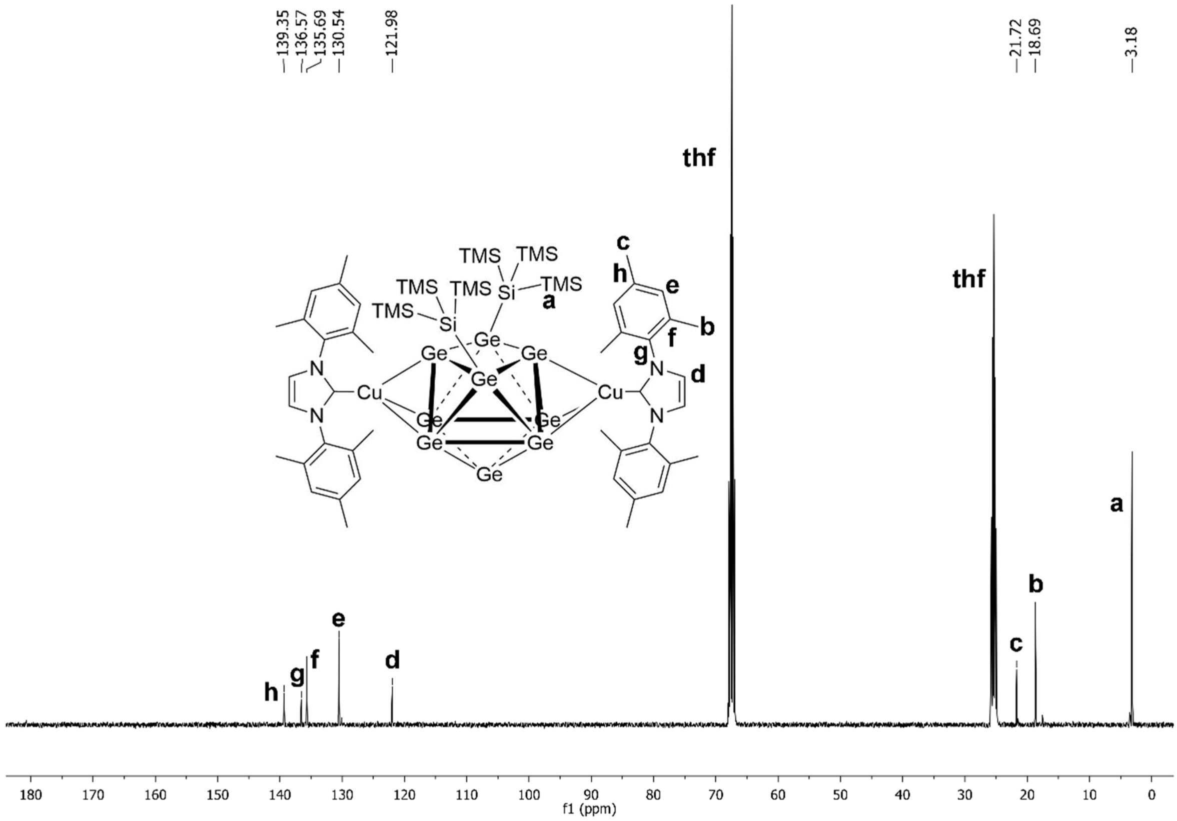

Figure SI 17: ^{13}C NMR of compound **5** in thf-d_8.

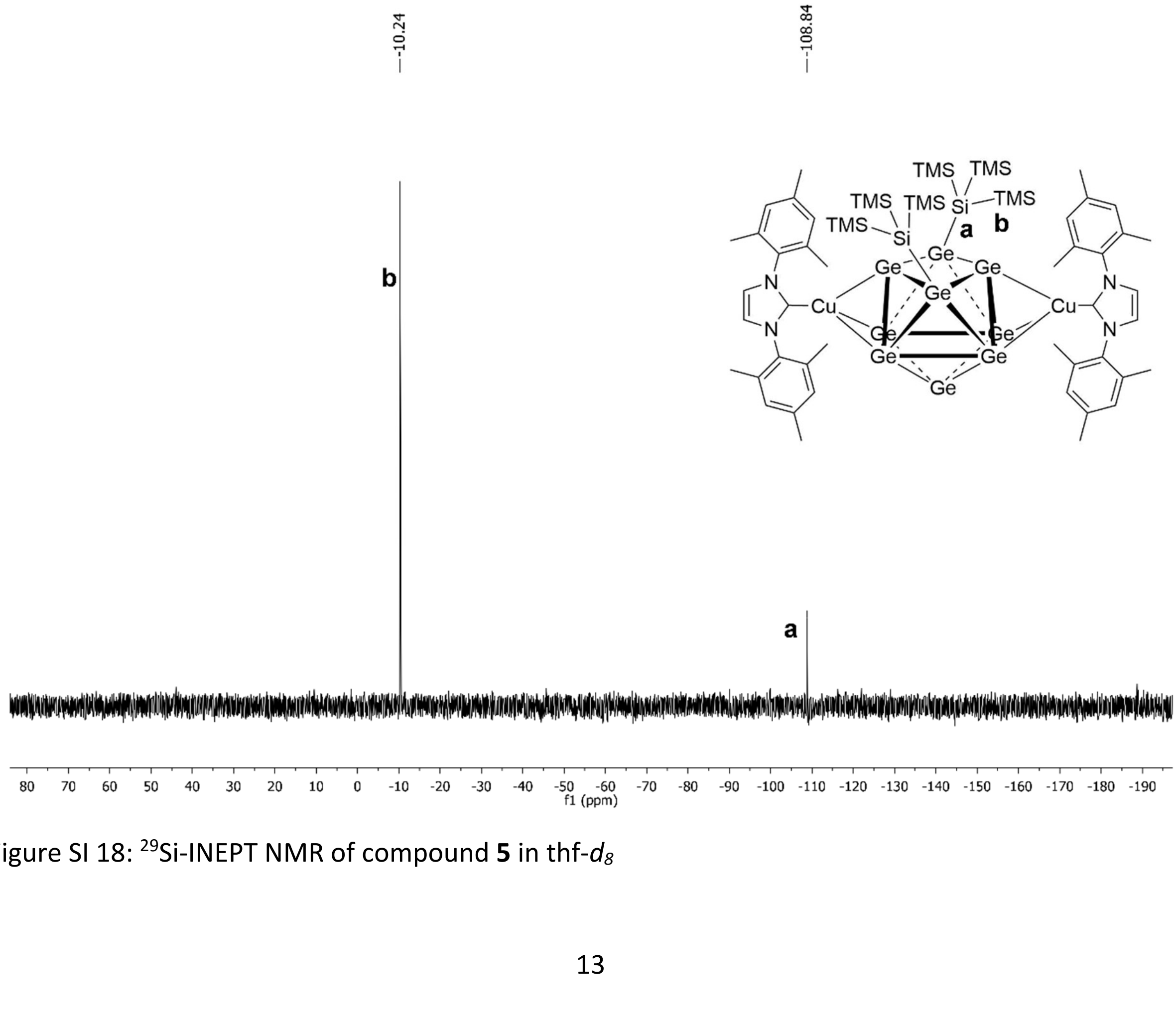

Figure SI 18: ^{29}Si-INEPT NMR of compound **5** in thf-d_8

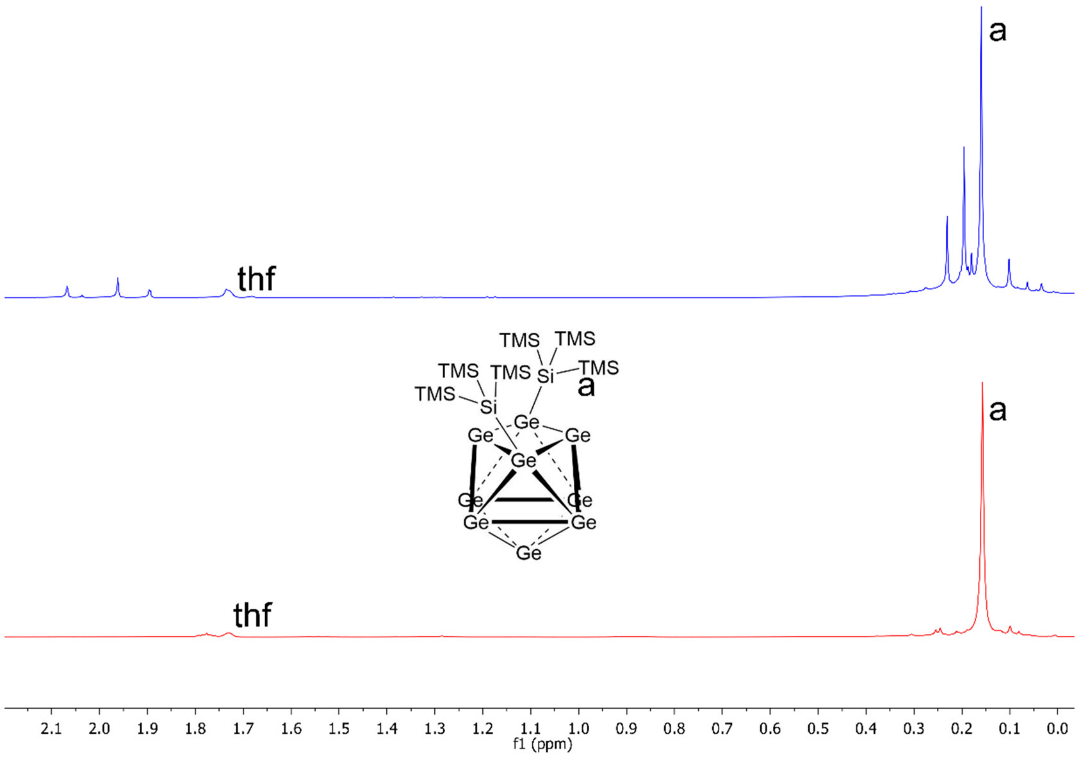

Figure SI 19: Comparison of ^{1}H NMR spectra of worked-up product of silylation of $K_{12}Ge_{17}$ with $Si(TMS)_3Cl$ (6 eq.) (blue/top) and $[Ge\{Si(TMS)_3\}]^{2-}$ (red/bottom) in thf-d_8.

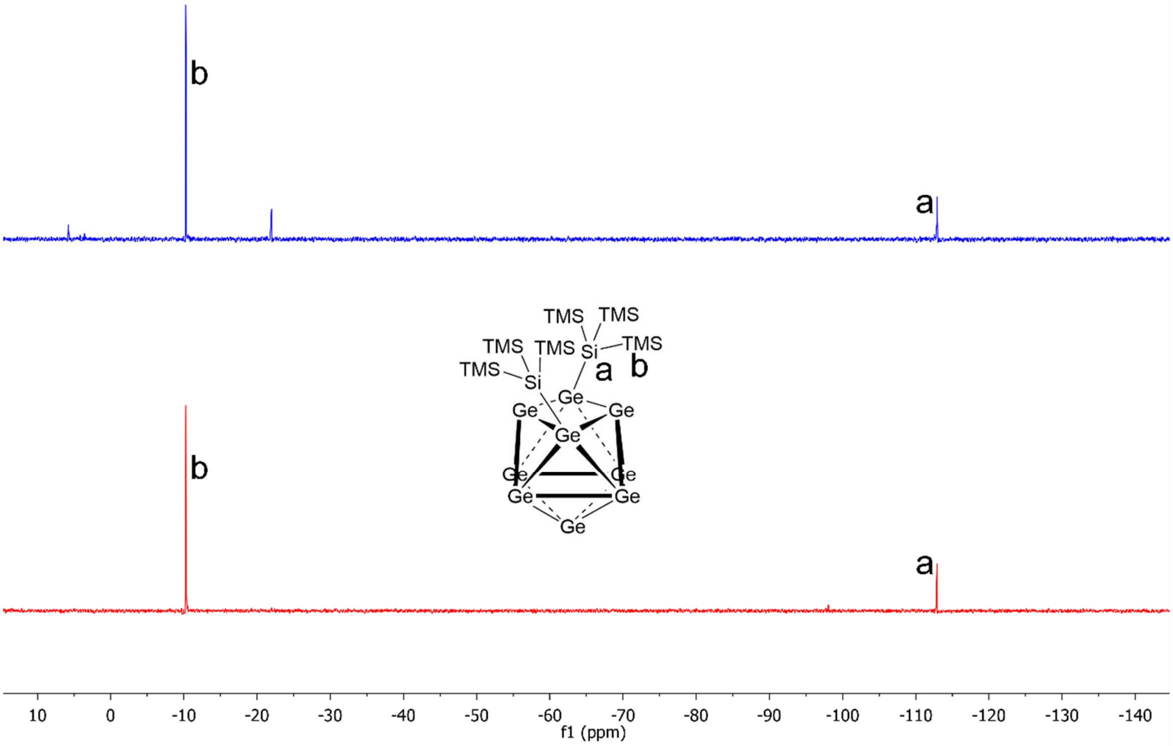

Figure SI 20: Comparison of ^{29}Si-INEPT NMR spectra of worked-up product of silylation of $K_{12}Ge_{17}$ with $Si(TMS)_3Cl$ (6 eq.) (blue/top) and $[Ge\{Si(TMS)_3\}]^{2-}$ (red/bottom) in thf-d_8.
